# UCHL1 stabilizes Twist1 via K11/K63-linked deubiquitination to drive tumor metastasis in non-small cell lung cancer

**DOI:** 10.1038/s41420-025-02925-8

**Published:** 2025-12-30

**Authors:** Qin Feng, Qianfang Hu, Qinghe Huang, Jingxing Yang, Ying Zhu, Feng Wang, Jianyu Xu, Sha Hu, Rujuan Zheng, Hui Shi, Zengyan Zhu, Xinyuan Ding, Wenjuan Wang

**Affiliations:** 1 Department of Pharmacy, Children’s Hospital of Soochow University, Suzhou, China; 2 Department of Pharmacy, Medical Science and Technology Innovation Center, The Affiliated Suzhou Hospital of Nanjing Medical University, Suzhou Municipal Hospital, Gusu School of Nanjing Medical University, Suzhou, China; 3 National Key Laboratory of Immunity and Inflammation, Suzhou Institute of Systems Medicine, Chinese Academy of Medical Sciences & Peking Union Medical College, Suzhou, China

**Keywords:** Ubiquitylation, Prognostic markers, Lung cancer

## Abstract

Deubiquitinating enzymes (DUBs) are critical regulators of protein turnover and have emerged as key players in cancer progression. In this study, we demonstrated that ubiquitin C-terminal hydrolase L1 (UCHL1) is upregulated in non-small cell lung cancer (NSCLC) and drives tumor metastatic progression, and we identified Twist1, a transcription factor that governs epithelial–mesenchymal transition (EMT), as a downstream target of UCHL1. Depletion of UCHL1 attenuated Twist1-mediated metastatic capacity in NSCLC cells both in vitro and in vivo. Mechanistically, UCHL1 directly interacts with Twist1 and stabilizes Twist1 protein levels through the enzymatic cleavage of K11- and K63-linked ubiquitin chains. Clinically, immunohistochemistry of human NSCLC tissues revealed a positive correlation between UCHL1/Twist1 expression and metastatic progression, with elevated levels of both proteins predicting poor prognosis. Our findings reveal a critical pathway through which UCHL1-mediated deubiquitination sustains Twist1 stability, revealing a novel posttranslational regulatory axis involved in cancer metastasis and progression and highlighting promising therapeutic targets for metastatic NSCLC.

## Introduction

Non-small cell lung cancer (NSCLC), accounting for approximately 85% of all lung cancer cases, represents the most prevalent form of the disease and is characterized by a high mortality rate due to its intricate pathogenic mechanisms [1]. Despite advancements in treatment modalities such as chemotherapy and radiation therapy, the five-year overall survival rate of patients with NSCLC remains at approximately 15% [2]. The progression of metastasis, a critical hallmark of advanced NSCLC, is closely linked to poor prognosis and a significantly reduced survival rate [3]. Therefore, deciphering the molecular intricacies driving NSCLC metastasis and pinpointing potential therapeutic targets within aberrant oncogenic pathways are urgently needed.

Initially recognized for its pivotal role in embryonic development, epithelial‒mesenchymal transition (EMT) is a reversible process by which differentiated epithelial cells undergo reprogramming to adopt a mesenchymal-like phenotype [4]. EMT is regulated by EMT-related transcription factors (EMT-TFs), which are activated by cytokine and growth factor networks within the tumor microenvironment (TME) during tumor progression [5]. Accumulating evidence underscores the role of EMT and its associated EMT-TFs in mediating cancer stem cell-like properties, chemoresistance, and metastatic spread [6–8]. Twist1, a key transcription factor in EMT, has been identified as a critical driver of metastasis and therapy resistance in NSCLC [9]. Compared with normal tissues, Twist1 is significantly overexpressed in NSCLC and other aggressive malignancies, with its upregulation associated with advanced tumor stage, increased metastatic burden, and unfavorable clinical outcomes [10, 11]. Therefore, targeting Twist1 holds promise as a strategic therapeutic approach for the management of NSCLC.

Twist1 expression is tightly regulated at both the transcriptional and posttranscriptional levels. In the TME, cytokines and growth factors such as IL-6, transforming growth factor-beta1 (TGF-β1), and epidermal growth factor (EGF) transcriptionally upregulate Twist1, leading to the repression of E-cadherin and the induction of EMT in cancer cells [12, 13]. Conversely, the protein turnover and activation of Twist1 are controlled by posttranslational modifications, notably ubiquitination. E3 ubiquitin ligases, including F-box and leucine-rich repeat protein 14 (FBXL14), beta-transducin repeat-containing protein (β-TrCP), and ring finger protein 8 (RNF8), play pivotal roles in the ubiquitination of Twist1, thereby affecting its degradation or activation [14–16]. However, the precise mechanisms underlying Twist1 stabilization in NSCLC remain unclear. Elucidating these mechanisms is expected to provide crucial insights for the development of therapeutic strategies targeting NSCLC and its metastatic progression.

Deubiquitinating enzymes (DUBs) constitute a critical regulatory axis within the ubiquitin‒proteasome system (UPS), specializing in the precise removal of ubiquitin moieties from substrate proteins to modulate their stability and function [17]. Accumulating evidence has established DUB dysregulation as a hallmark of carcinogenesis, with particular implications for metastatic progression across malignancies [18, 19]. The mechanistic involvement of specific DUBs in metastatic cascades is becoming increasingly apparent. For instance, ubiquitin-specific protease 12 (USP12) and BRCA1/BRCA2-containing complex subunit 3 (BRCC3) facilitate esophageal squamous cell carcinoma dissemination via the stabilization of metastatic effector proteins [20, 21]. Our prior work revealed that ubiquitin-specific protease 5 (USP5) promotes NSCLC metastasis through direct EMT induction, further substantiating the DUB–metastasis axis [22]. Additional deubiquitinating enzymes, including ubiquitin-specific peptidase 26 (USP26) and OTU domain-containing ubiquitin aldehyde-binding protein 1 (OTUB1), promote the metastasis of esophageal squamous cell carcinoma by stabilizing Snail, another EMT transcription factor [23, 24].

Notably, ubiquitin carboxyl-terminal hydrolase L1 (UCHL1) represents a bifunctional DUB family member that orchestrates diverse oncogenic pathways through context-dependent substrate deubiquitination [25–27]. Although conventionally associated with pro-survival and migratory phenotypes, UCHL1 demonstrates paradoxical EMT-suppressive activity in cadmium-transformed bronchial epithelia [28]. Intriguingly, despite being differentially expressed in NSCLC versus normal pulmonary tissue, the precise role of UCHL1 in metastatic regulation remains enigmatic. This apparent contradiction between its established regulatory capacity and unresolved functionality in NSCLC progression underscores the need for mechanistic investigation.

Our findings demonstrated that elevated expression of UCHL1 significantly enhances the migration and promotes the EMT of NSCLC cells. Furthermore, our research identifies UCHL1 as a bona fide DUB for Twist1. By directly binding to Twist1 and subsequently deubiquitinating it, UCHL1 stabilizes this protein, thereby facilitating Twist1-mediated tumor metastasis in NSCLC through the induction of EMT. Collectively, the results of our study elucidate the critical role of the UCHL1/Twist1 axis as a pivotal regulatory mechanism that governs EMT and metastasis, suggesting the development of innovative targeted therapies for the treatment of NSCLC.

## Results

### UCHL1 is upregulated in human NSCLC tissues and correlates with poor prognosis

**Fig. 1:**
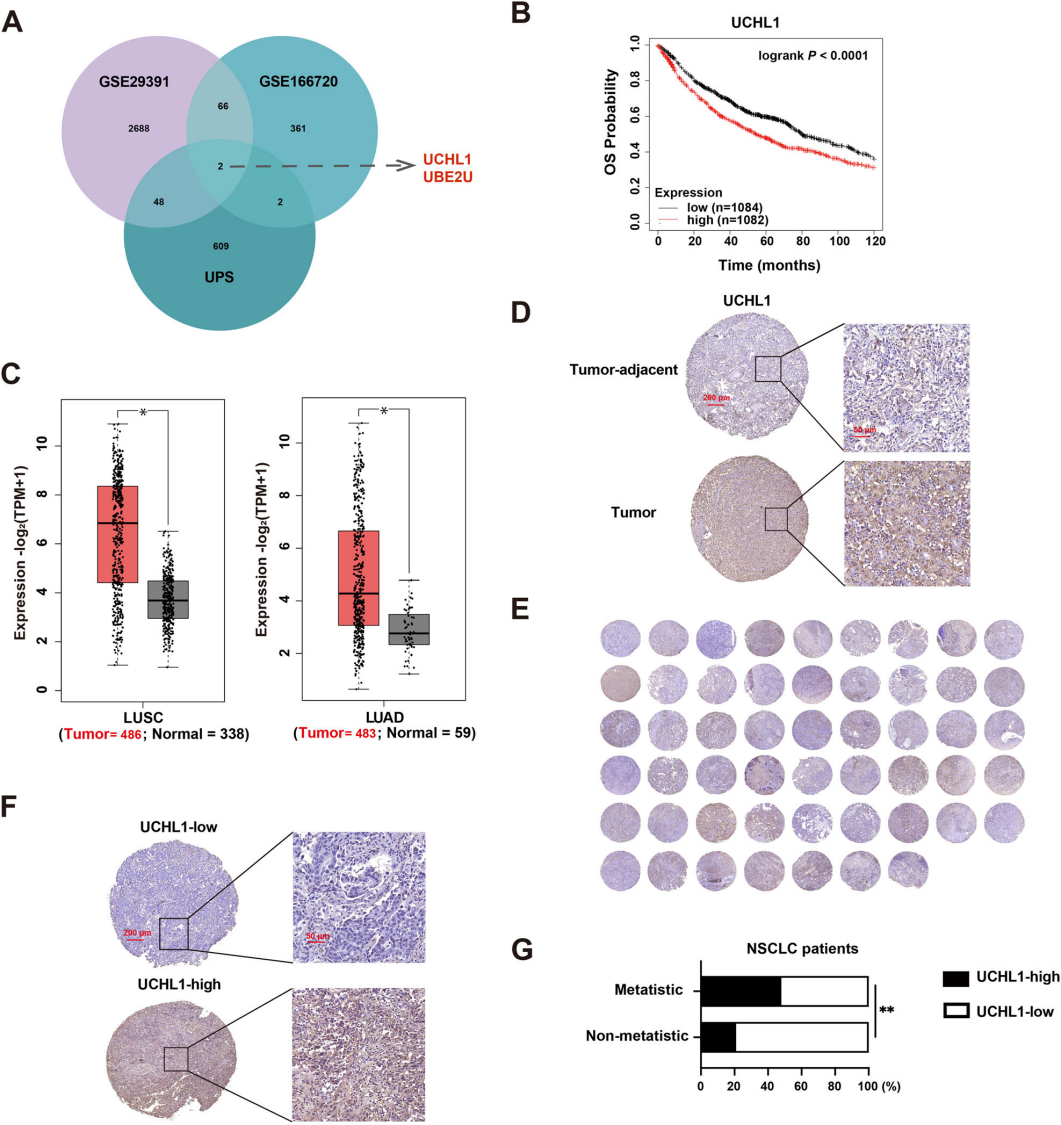
UCHL1 is highly expressed in human NSCLC tissues and is correlated with poor prognosis. **A** Members of the DUB family were found to be positively associated with NSCLC metastasis. **B** Overall survival (OS) was analyzed based on the expression of *UCHL* in NSCLC samples using Kaplan–Meier plotter. **C** Box plots were generated to compare *UCHL1* mRNA levels between lung adenocarcinoma (LUAD, *n* = 483) and normal lung tissues (*n* = 59), and between lung squamous cell carcinoma (LUSC, *n* = 486) and normal lung tissues (*n* = 338) from the TCGA database. Log-rank test. **D** Representative images of UCHL1 immunohistochemical staining in tumor-adjacent and tumor tissues are shown. **E** UCHL1 expression was assessed in a tissue microarray (TMA) consisting of 52 NSCLC samples. **F** Representative IHC images of UCHL1 expression in metastatic and nonmetastatic tissues are presented. **G** The correlation between different UCHL1 expression levels and metastasis status in 52 NSCLC samples was analyzed. Chi-square test. *p < 0.05, **p < 0.01.

Previous research has underscored the critical role of DUBs in the metastatic processes of cancer [29]. To investigate potential DUBs involved in NSCLC metastasis, we conducted a systematic analysis of GEO datasets (GSE29391 and GSE166720) and 661 ubiquitin‒proteasome system-related genes to identify genes that are differentially expressed in NSCLC. This screening identified the deubiquitinating enzyme UCHL1 and the E2 ubiquitin ligase ubiquitin-conjugating enzyme E2 U (UBE2U) as metastasis-associated genes whose expression profiles are significantly altered in NSCLC (Fig. 1A). Notably, high UCHL1 expression correlated with adverse clinical outcomes in patients with NSCLC, whereas UBE2U expression did not significantly affect prognosis (Figs. 1B and S1). TCGA database analysis confirmed substantial UCHL1 upregulation in NSCLC tissues compared with adjacent normal lung tissue (Fig. 1C). Consistent with these findings, immunohistochemical analysis revealed higher UCHL1 protein levels in human NSCLC tissues than in adjacent normal tissues (Fig. 1D). In our clinical cohort of 52 patients with NSCLC, immunohistochemical scoring (IHC score cutoff = 3) stratified the patients into UCHL1-high (*n* = 17) and UCHL1-low (*n* = 35) subgroups (Fig. 1E, F). Notably, UCHL1 overexpression was significantly positively correlated with metastatic progression (Fig. 1G), suggesting its potential role as a molecular driver of NSCLC metastasis.

### High UCHL1 expression was positively correlated with EMT and the migration of NSCLC cells

**Fig. 2:**
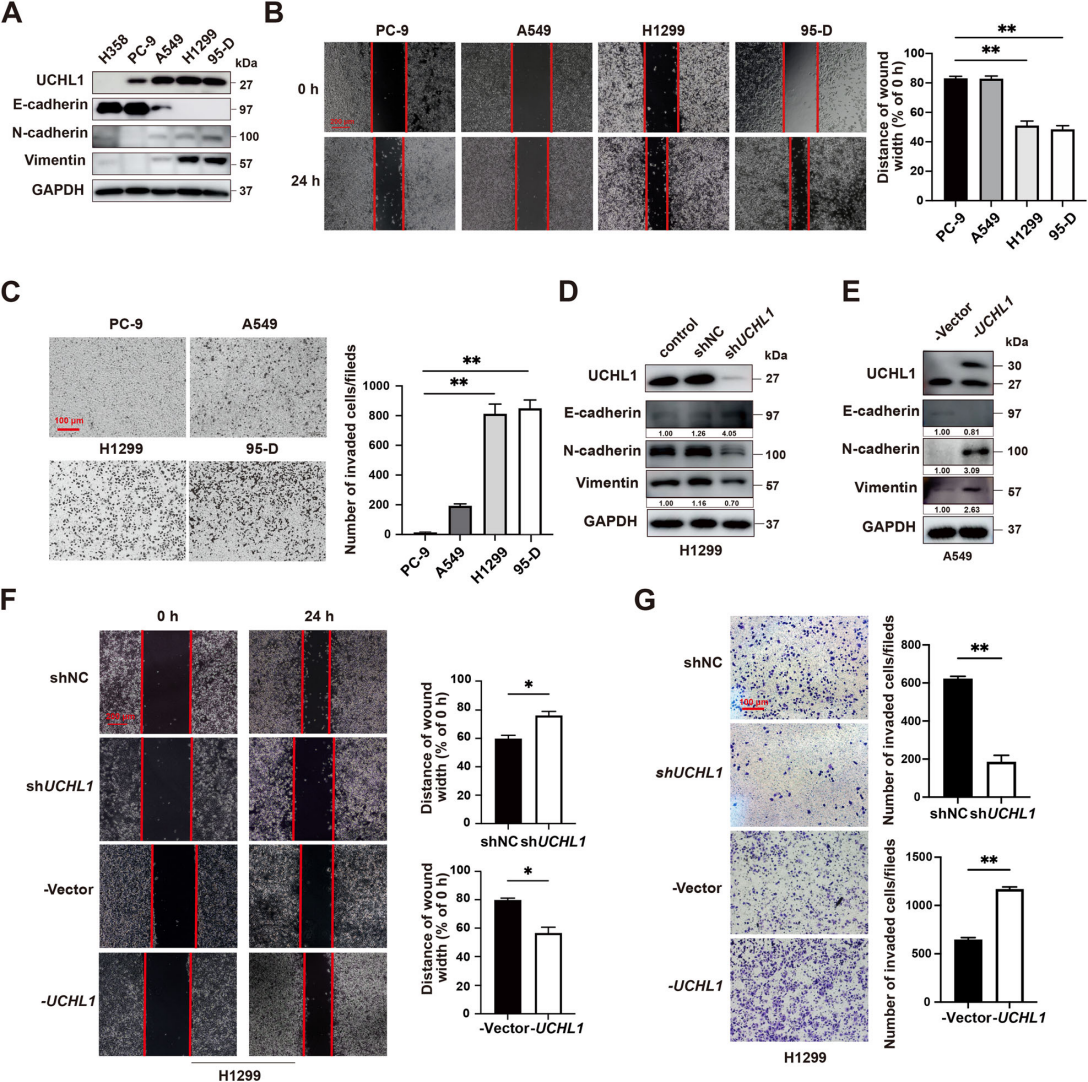
High UCHL1 expression is positively correlated with EMT and migration in NSCLC cells. **A** Western blot analysis was performed to assess UCHL1 and EMT marker expression in NSCLC cell lines. **B** The migration of NSCLC cells was evaluated using a wound-healing assay. Red bar, 200 μm. **C** Cell invasion was measured in a transwell invasion assay. Red bar, 100 μm. **D** EMT marker protein levels were analyzed in H1299 cells treated with sh*UCHL1* or shNC lentivirus by western blotting. **E** EMT marker protein levels were assessed in A549 cells transfected with Vector or Flag-*UCHL1* plasmids by western blotting. **F** Cell migration was determined using wound-healing assays. Red bar, 200 μm. **G** Cell invasion was evaluated in a transwell invasion assay. Red bar, 100 μm. In (**B**, **C**, **F**, and **G**), Mann–Whitney test (*n* = 3, independent experiments), *p < 0.05, **p < 0.01.

To investigate the role of UCHL1 in NSCLC metastasis, we first analyzed its expression across various NSCLC cell lines. UCHL1 levels were significantly higher in H1299 and 95-D cells than in H358, PC-9, and A549 cells. Concurrently, H1299 and 95-D cells exhibited a more pronounced EMT profile, as evidenced by a reduction in the epithelial marker E-cadherin and an increase in the mesenchymal markers N-cadherin and Vimentin at the protein level (Fig. 2A). The results of wound-healing and Transwell invasion assays corroborated this molecular profile, revealing greater migration and invasion capacities in H1299 and 95-D cells than in low-UCHL1 PC-9 control cells (Fig. 2B, C). Lentiviral knockdown or overexpression of UCHL1 demonstrated that UCHL1 knockdown in H1299 cells reversed EMT progression, increased E-cadherin expression, and decreased N-cadherin and Vimentin expression, whereas UCHL1 overexpression in A549 and PC-9 cells promoted EMT progression (Fig. 2D, E, and Fig. S2A). To assess the functional impact of UCHL1 on cell migration and invasion, we conducted wound-healing and Transwell assays in H1299 and PC-9 cells. UCHL1 overexpression increased the migration and invasion capacities of H1299 and PC-9 cells, whereas UCHL1 knockdown suppressed the migration and invasion capacities of H1299 cells (Fig. 2F, G, and Fig. S2B, C). Collectively, these findings confirm that UCHL1 plays a pivotal role in promoting EMT, migration, and invasion of NSCLC cells.

### UCHL1 interacts with Twist1 and upregulates Twist1 protein levels

**Fig. 3:**
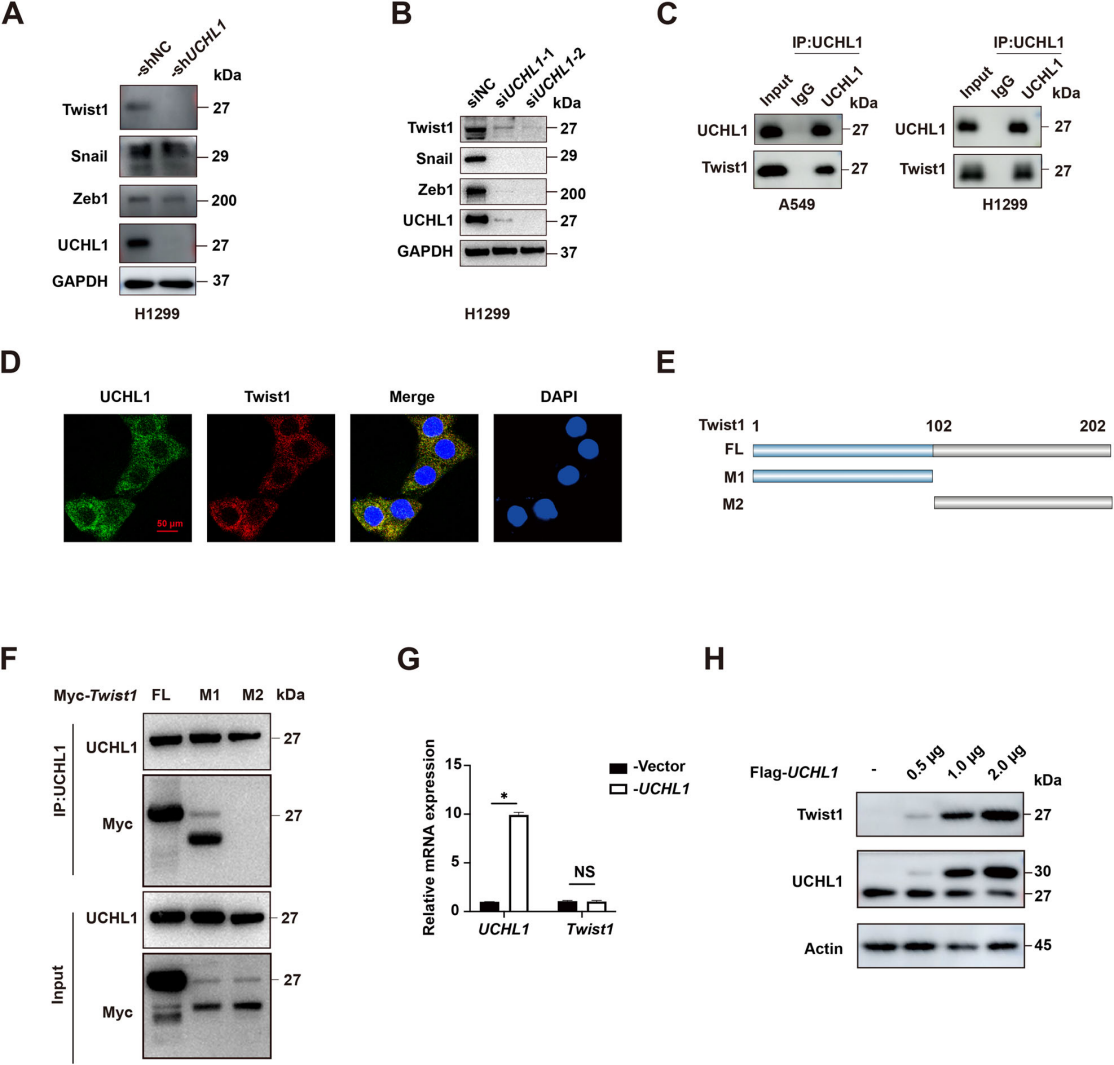
UCHL1 interacts with Twist1 and positively regulates Twist1 protein levels. **A** EMT-TF protein levels were analyzed in H1299 cells treated with sh*UCHL1* or shNC lentivirus by western blotting. **B** EMT-TF protein levels were assessed in H1299 cells treated with si*UCHL1* or siNC by western blotting. **C** Cell lysates were subjected to immunoprecipitation with control IgG, anti-UCHL1, and anti-Twist1 antibodies. **D** Double staining for UCHL1 and Twist1 in NSCLC cells was visualized by confocal microscopy, with nuclei counterstained with DAPI. Red bars, 50 μm. **E** Overview of Twist1 structures. **F** HEK293T cells transfected with the indicated constructs were subjected to immunoprecipitation with anti-Myc and anti-UCHL1 antibodies. **G** Changes in *Twist1* mRNA levels in H1299 cells transfected with Flag-*UCHL1* plasmids were analyzed. Mann–Whitney test (*n* = 3, independent experiments). **H** Changes in Twist1 protein levels in H1299 cells transfected with different concentrations of Flag-*UCHL1* plasmids were analyzed by western blotting. *p < 0.05, *NS* not significant.

Mechanistic investigations into UCHL1-mediated NSCLC metastasis revealed the critical regulation of EMT transcription factors (EMT-TFs). Our data revealed that UCHL1 knockdown downregulated Twist1, Snail, and Zeb1 expression, underscoring the pivotal role of UCHL1 in the EMT process (Fig. 3A, B). Among these EMT-TFs, only Twist1 was confirmed to interact with UCHL1 by coimmunoprecipitation (Co-IP) analysis, which validated their direct physical interaction in A549 and H1299 cells (Fig. 3C). This interaction was further substantiated by confocal laser scanning microscopy, which revealed significant colocalization of UCHL1 and Twist1 in NSCLC cells (Fig. 3D). These results collectively confirm that UCHL1 interacts with Twist1 in NSCLC cells.

To identify the specific region of Twist1 that interacts with UCHL1, we generated two truncated fragments of Twist1 that collectively cover its full length (Fig. 3E). Co-IP assays demonstrated that the M1 region is essential for the UCHL1–Twist1 interaction (Fig. 3F). These data suggest that UCHL1 physically interacts with Twist1. Given that UCHL1 is a deubiquitinase, we hypothesized that it may regulate the protein stability of Twist1. Indeed, overexpression of UCHL1 resulted in increased Twist1 protein levels without affecting its mRNA levels (Fig. 3G), suggesting that UCHL1 does not influence Twist1 transcription. Furthermore, UCHL1 overexpression increased Twist1 protein levels in a dose-dependent manner (Fig. 3H). These findings indicate that UCHL1 modulates the expression of Twist1 at the posttranscriptional level, likely through its deubiquitinating activity and subsequent stabilization of the Twist1 protein.

### UCHL1 removes K11/K63-linked ubiquitin chains from Twist1 through its deubiquitinase activity

**Fig. 4:**
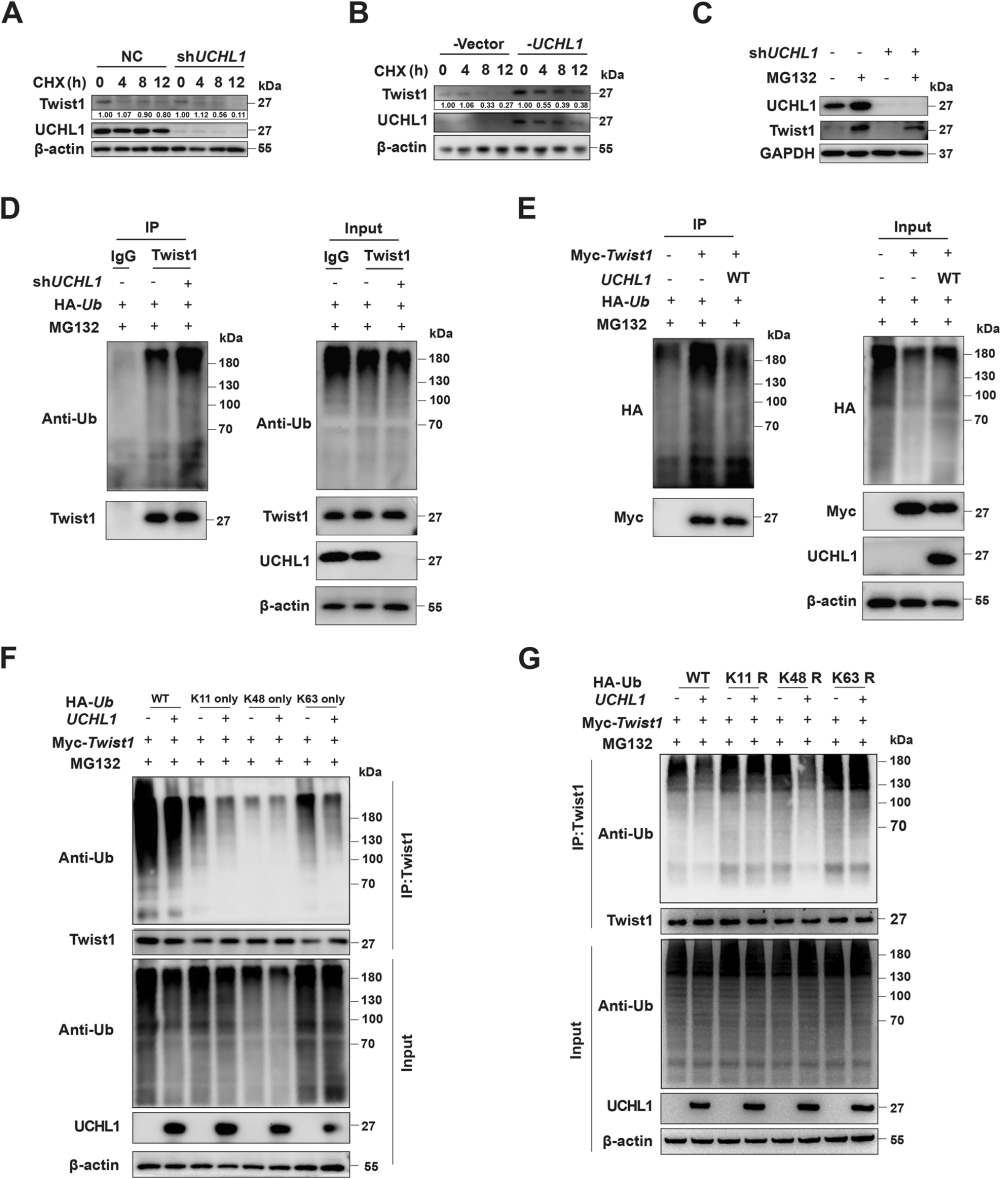
UCHL1 stabilizes Twist1 through its deubiquitinase activity. **A** H1299 cells line transfected with shRNAs was exposed to 20 μg/mL cycloheximide (CHX) for various durations, and the stability of the Twist1 protein was assessed by western blotting. **B** Following transfection with Flag-Vector or Flag-*UCHL1* for 24 h, H1299 cells were treated with CHX at 20 μg/mL for different durations to evaluate Twist1 protein stability by western blotting. **C** H1299 cells transfected with shRNAs were incubated with MG132 at 20 μM for 10 h before being harvested, and the level of Twist1 protein was determined by western blotting. **D** H1299 cells transfected with shRNAs and HA-Ubiquitin were treated with 20 μM MG132 for 10 h, and the ubiquitination status of endogenous Twist1 was determined using a ubiquitination assay. **E** HEK293T cells cotransfected with Myc-*Twist1*, HA-Ub, and UCHL1-WT constructs were treated with 20 μM MG132 for 8 h, and the ubiquitination level of Myc-Twist1 was assessed using ubiquitination assays. **F** Examination of the influence of UCHL1 on specific ubiquitination types of Twist1. HEK293T cells cotransfected with the indicated plasmids were treated with 20 μM MG132 for 8 h, and the level of ubiquitinated Myc-*Twist1* was measured using ubiquitination assays. **G** Examination of the influence of UCHL1 on specific ubiquitination types of Twist1. HEK293T cells that were cotransfected with the indicated plasmids were treated with 20 μM MG132 for 8 h, and the level of ubiquitinated Myc-*Twist1* was measured using ubiquitination assays. WT wild type, K Lysine, R Arginine. All the data are representative of three independent experiments. *p < 0.05.

To further investigate the mechanism by which UCHL1 regulates Twist1 stability via deubiquitination, we evaluated the half-life of Twist1 protein in cells subjected to UCHL1 knockdown or overexpression in the presence of the protein translation inhibitor cycloheximide (CHX). Our findings indicated that Twist1 protein stability was compromised in the absence of UCHL1, whereas UCHL1 overexpression significantly increased Twist1 stability (Fig. 4A, B, and S3A). Additionally, the proteasome inhibitor MG132 reversed the reduction in Twist1 levels caused by UCHL1 knockdown, suggesting that Twist1 degradation was mediated by the proteasome pathway (Fig. 4C).

Further ubiquitination assays were performed to examine Twist1 ubiquitination levels in the presence of the proteasome inhibitor MG132. As expected, UCHL1 knockdown dramatically increased Twist1 ubiquitination (Fig. 4D). Importantly, wild-type (WT) UCHL1, but not the catalytically inactive C90S mutant, efficiently removed ubiquitin chains from Twist1 (Fig. 4E and Fig. S3B). We next evaluated the effect of UCHL1 on the specific ubiquitination of Twist1. To this end, we cotransfected Twist1 with hemagglutinin (HA)-tagged ubiquitin mutants. Co-IP assays revealed that UCHL1 dramatically removed K11- and K63-linked ubiquitin chains from Twist1 (Fig. 4F). Consistent with these findings, when Twist1 was cotransfected with HA-ubiquitin mutants in which a single lysine residue was mutated to arginine (KR), K11R- and K63R-linked ubiquitination signals on Twist1 were not removed from UCHL1 (Fig. 4G). These results indicate that UCHL1 predominantly cleaves K11- and K63-linked ubiquitin chains from Twist1. Collectively, these findings confirm that UCHL1 plays a role in maintaining Twist1 stability by cleaving the K11- and K63-linked ubiquitin chains of Twist1 through its deubiquitinase activity.

### UCHL1 promotes NSCLC cells' EMT and migration by regulating Twist1 protein levels

**Fig. 5:**
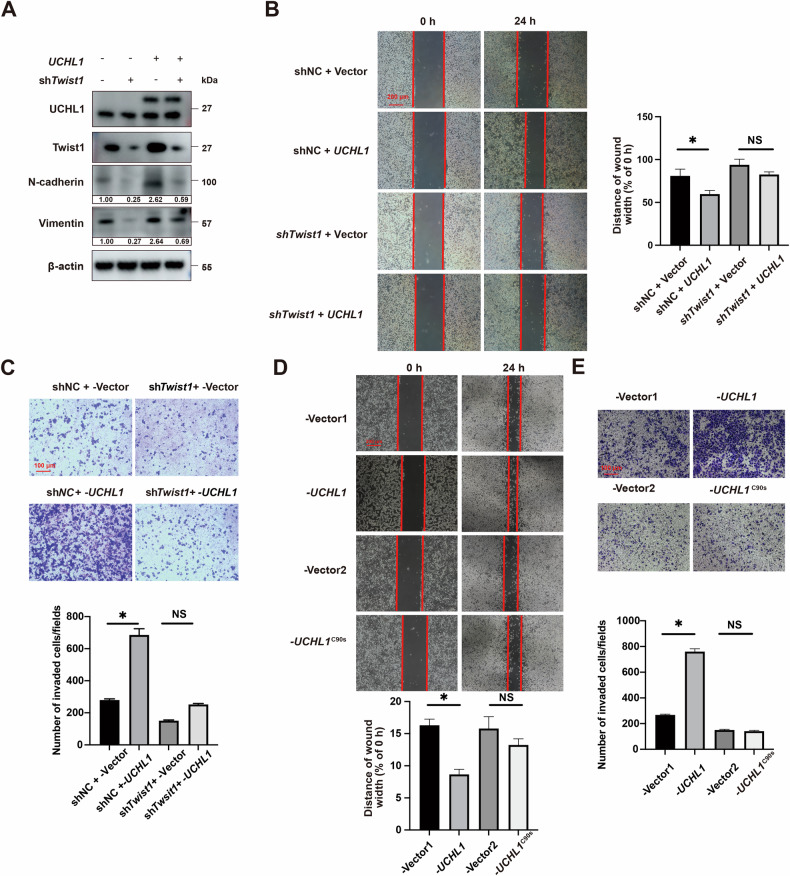
UCHL1 upregulates Twist1 expression to promote NSCLC cell EMT and migration in vitro. UCHL1 was introduced into H1299 cells transduced with *Twist1* shRNA. **A** The expression levels of E-cadherin, N-cadherin, and Vimentin were analyzed by western blotting to assess the effects of UCHL1 on the expression of EMT markers. **B** Wound-healing assays were performed to determine cell migration when H1299 cells were transfected with shNC + Vector, shNC + *UCHL1*, sh*Twist1* + Vector, or sh*Twist1* + *UCHL1*. Red bars, 200 μm. **C** Transwell invasion assays were performed to measure cell migration when H1299 cells were transfected with shNC + Vector, shNC + *UCHL1*, sh*Twist1* + Vector, or sh*Twist1* + *UCHL1*. Red bars. 100 μm. **D** Wound-healing assays were performed to determine cell migration when H1299 cells were transfected with Flag-Vector, Flag-*UCHL1*, or Flag-*UCHL1*^C90S^. Red bars, 200 μm. **E** Transwell invasion assays were used to measure cell migration when H1299 cells were transfected with Flag-Vector, Flag-*UCHL1*, or Flag-*UCHL1*^C90S^. Red bars, 100 μm. In (**B**, **C**, **D**, **E**), Mann–Whitney test, (*n* = 3, independent experiments), *p < 0.05, NS not significant.

In our previous work, we demonstrated that UCHL1 knockdown inhibits the EMT and migration of NSCLC cells. In this study, we further investigated the effects of UCHL1 on Twist1-induced EMT [9] and the migration of NSCLC cells. Western blot analysis of H1299 cells overexpressing UCHL1 revealed increased levels of Vimentin and N-cadherin, indicating EMT induction. However, this induction was reversed by Twist1 knockdown (Fig. 5A). Cell migration and invasion were assessed using wound-healing and transwell assays. The wound-healing assay demonstrated that UCHL1 overexpression promoted the migration of H1299 cells, and this enhancement was reversed by concomitant Twist1 knockdown (Fig. 5B). Similarly, the results of the transwell assay revealed that UCHL1 overexpression increased the number of transmembrane H1299 cells, an effect that was also abolished by Twist1 knockdown (Fig. 5C). Consistent with its role in stabilizing Twist1, wound-healing and transwell assays revealed that WT UCHL1, but not the catalytically inactive C90S mutant, was capable of inducing the migration and invasion of NSCLC cells (Fig. 5D, E). Collectively, these findings suggest that UCHL1 promotes EMT, migration, and invasion in NSCLC cells by stabilizing Twist1, with its catalytic activity being critical for this metastasis-promoting function.

### UCHL1 promotes the metastasis of NSCLC cells in vivo

**Fig. 6:**
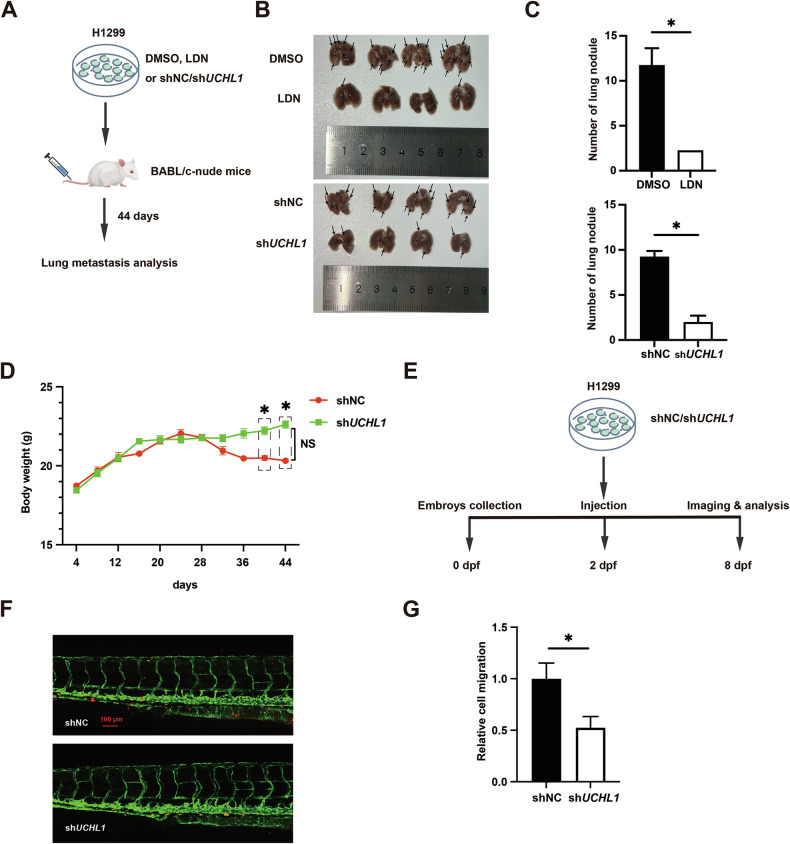
UCHL1 promotes NSCLC cell EMT and migration in vivo. **A**–**C** Lung metastasis nodule quantification and representative images after tail-vein injection of LDN- and UCHL1-knockdown H1299 cells (*n* = 4 mice per group). **D** Body weights were evaluated using analysis of variance (*n* = 4). **E**, **F** Analysis of invasive cell numbers in NSCLC cells pretreated with shNC or sh*UCHL1* in zebrafish metastasis experiments. Dpf, day post-fertilization. Red bars, 100 μm. The error bars represent the mean ± SEM from at least three independent experiments. In (**C**, **D**, **G**), Mann–Whitney test, *p < 0.05.

To explore the in vivo effects of the UCHL1–Twist1 axis on NSCLC metastasis, we generated H1299 cells with stable UCHL1 knockdown using lentiviral transfection. We then used a tail-vein injection model in BABL/c-nude mice to assess lung metastasis and compared control cells with UCHL1-knockdown cells. After 44 days, the mice were euthanized, and lung metastatic colonies were quantified (Fig. 6A, B), which revealed that the number of nodules on the surface of lung tissue in the LDN group was significantly lower than that in the DMSO group. Consistent with these findings, UCHL1 knockdown significantly reduced the number of lung surface nodules in mice (Fig. 6C), and this marked reduction in tumor burden was accompanied by increased body weight in the knockdown group at 31 days post-injection, suggesting that UCHL1 may influence the overall health of the mice (Fig. 6D).

To further elucidate the role of UCHL1 activity in tumor metastasis, we examined the impact of UCHL1 misexpression on cell extravasation using a zebrafish xenograft model of lung cancer. The results demonstrated that UCHL1 knockdown not only reduced invasive ability but also diminished the metastatic phenotype, with cells failing to extravasate into the zebrafish tail fin and instead forming clusters around the blood vessels (Fig. 6E–G). Furthermore, cellular immunofluorescence revealed that UCHL1 overexpression in PC-9 cells resulted in a marked increase in Ki67 expression, indicating that elevated UCHL1 levels promote proliferative activity in NSCLC cells (Fig. S4). Taken together, these findings suggest that UCHL1 knockdown significantly reduces NSCLC metastasis by impairing cell invasive ability.

### The overexpression of UCHL1 and Twist1 is positively correlated with metastasis and poor prognosis in NSCLC

**Fig. 7:**
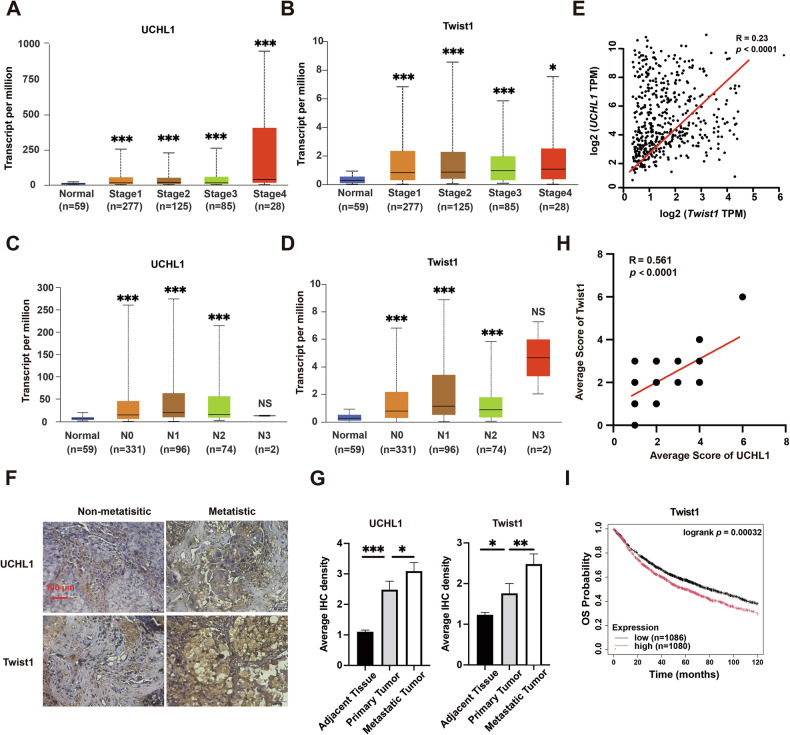
Dysfunction of UCHL1 and Twist1 is correlated with poor prognosis in patients with NSCLC. **A**, **B** Relationships of *UCHL1* and *Twist1* expression with LUAD stages according to data from the TCGA database. **C**, **D** Relationships of *UCHL1* and *Twist1* expression with the lymph node metastasis status of patients with LUAD according to data from the TCGA database. N0: no regional lymph node metastasis; N1: 1–3 axillary lymph node metastases; N2: 4–9 axillary lymph node metastases; N3: >9 axillary lymph node metastases. Data are shown as box plots with medians, interquartile ranges, and lower/upper whiskers in (**A** and **B**). **E** Positive correlation between *UCHL1* and *Twist1* expression in lung tissues according to data from the TCGA database. Pearson’s correlation analysis was performed to determine correlation coefficients and *p*-values. **F**, **G** Representative and quantitative IHC of UCHL1 and Twist1 in LUAD tissue arrays containing primary lung tumors with paired adjacent normal lung tissues and long-distance metastatic tumors from primary LUAD. Red bars, 100 μm. Mann–Whitney test. **H** Linear regression analysis and Spearman’s correlation were used to determine the relationship between UCHL1 and Twist1 protein expression in NSCLC tissue specimens. **I** *Twist1* positively correlates with poor prognosis in human NSCLC tissues. Overall survival (OS) was analyzed based on the expression of *Twist1* in NSCLC samples using the Kaplan–Meier plotter. *p < 0.05, **p < 0.01, ***p < 0.001, NS not significant.

To assess the clinical significance of UCHL1 and Twist1 in NSCLC progression, we analyzed datasets from the TCGA database (https://ualcan.path.uab.edu/analysis.html). We found that UCHL1 expression was elevated at all stages in lung adenocarcinoma (LUAD) tissues compared with normal lung tissues (Fig. 7A). Similarly, Twist1 was overexpressed across all LUAD stages (Fig. 7B). Additionally, compared with those from affected lymph nodes, primary lung tumors from patients without lymph node metastasis (N0) exhibited the lowest UCHL1 expression. Meanwhile, Twist1 expression in primary lung tumors increased with advancing nodal metastasis, with the exception of the N3 group, possibly due to the limited number of cases (Fig. 7C, D). A strong positive correlation was observed between *UCHL1* and *Twist1* levels in lung tissues (Fig. 7E), suggesting that these genes cooperate in lung cancer progression.

We further investigated UCHL1 and Twist1 expression in tumor samples and adjacent normal tissues from 52 patients with NSCLC and correlated these findings with clinicopathologic data. NSCLC tissues showed more intense immunostaining for both UCHL1 and Twist1, whereas nonmetastatic lung tissues exhibited weaker staining (Fig. 7F). Immunohistochemistry (IHC) confirmed significantly higher levels of UCHL1 and Twist1 in cancer tissues than in adjacent normal tissues (Fig. 7G). Moreover, higher UCHL1 expression correlated with stronger Twist1 expression in tumor tissues, as demonstrated by a significant positive correlation determined by Spearman’s rank correlation analysis (Fig. 7H). We also investigated the prognostic significance of this expression pattern for survival outcomes in patients with NSCLC. The results revealed that patients with high Twist1 expression had a poorer prognosis than those with low Twist1 expression (Fig. 7I). Collectively, these findings suggest that the UCHL1/Twist1 axis may serve as a potential prognostic marker and therapeutic target for NSCLC.

## Discussion

**Fig. 8:**
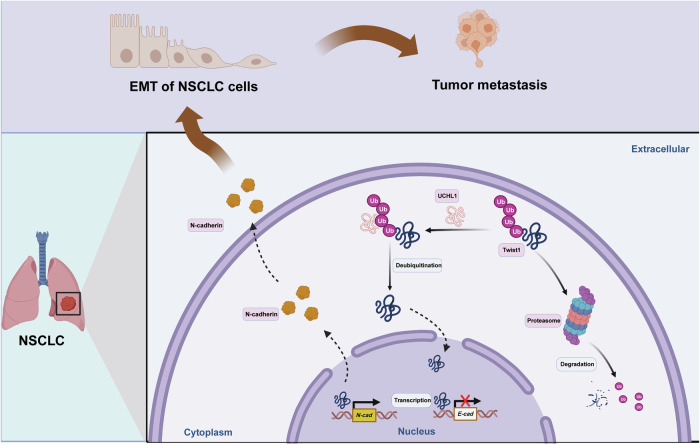
Schematic model illustrating the role of UCHL1 in Twist1 deubiquitination and the promoting effect of UCHL1/Twist1 on NSCLC progression. In NSCLC, highly expressed UCHL1 deubiquitylates Twist1 by removing K11/K63-linked ubiquitin chains, thereby stabilizing and upregulating the Twist1 protein. Increased Twist1 expression is involved in the nuclear transcription of N-cadherin but not E-cadherin, thereby promoting the epithelial‒mesenchymal transition (EMT) process and facilitating NSCLC metastasis.

Despite significant advances in the diagnosis and treatment of NSCLC, the therapeutic targets influencing its progression and the precise underlying mechanisms remain incompletely understood [30]. In our previous research, we reported a strong association between DUBs and NSCLC development [31]. However, the role of UCHL1 in NSCLC metastasis has not been previously investigated. Here, we observed a positive correlation between elevated UCHL1 expression and both EMT and the migration of NSCLC cells. Furthermore, we identified UCHL1 as a deubiquitinase for Twist1 that stabilizes Twist1 protein levels in NSCLC cells and thereby promotes EMT, invasion, and migration. Clinical significance and prognosis analysis revealed that high Twist1 expression serves as an additional risk factor for poor prognosis modulated by UCHL1 in patients with NSCLC. These results indicate that deubiquitination-mediated stabilization of Twist1 is a crucial mechanism through which UCHL1 drives NSCLC progression (as shown in Fig. 8). Moreover, our findings underscore the critical role of the UCHL1/Twist1 signaling pathway in NSCLC development, potentially offering novel therapeutic targets for clinical management.

Distant metastasis is a complex process involving EMT, a pivotal early event that significantly contributes to the detachment, migration, and invasion of cancer cells. EMT is characterized by the suppression of epithelial cell markers such as E-cadherin and claudin-1 and the concurrent promotion of mesenchymal cell markers, including N-cadherin and Vimentin [32]. Twist1, a transcription factor that induces EMT, is crucial for cancer development, progression, and the establishment of metastatic potential [33]. Its expression and stability are regulated at multiple levels, including posttranslational modifications by ubiquitination and deubiquitination [15]. For instance, the upregulation of Twist1 in NSCLC cells and tissues has been shown to induce EMT, thereby facilitating cancer metastasis [34]. Although the role of Twist1 in cancer metastasis is well documented, the mechanisms underlying its stabilization in NSCLC are not yet fully understood. In this study, we identified UCHL1 as a key upregulator of Twist1 protein levels in NSCLC, resulting in a marked increase in the mesenchymal markers Vimentin and N-cadherin and a corresponding decrease in the epithelial marker E-cadherin. Conversely, knockdown of Twist1 reversed these changes. Additionally, we demonstrated that UCHL1-mediated regulation of the EMT process is dependent on Twist1. These insights offer a novel perspective on the role of UCHL1 in mediating NSCLC metastasis through the modulation of EMT.

Twist1 acts as a key EMT-related transcription factor through direct transcriptional repression of E-cadherin and transcriptional activation of N-cadherin [35]. Twist1 expression is controlled at multiple levels. Under hypoxia, hypoxia-inducible factor 1α (HIF1α) upregulates Twist1 to induce EMT and metastatic dissemination [36]. MAP kinases (MAPKs) can phosphorylate Twist1, resulting in Twist1 protein stabilization [37]. Our recent work revealed that UCHL1 functions as a deubiquitinase to remove ubiquitin from Twist1, resulting in Twist1 protein stabilization. We further explored the intricate interactions and regulatory pathways between UCHL1 and Twist1. As a DUB, UCHL1 is known for its role in deubiquitinating a broad array of targets, influencing various cellular processes, and contributing to malignancies across different cancer types [27]. Previous work by Cheng et al. demonstrated that UCHL1 can increase the expression of lysine-specific demethylase 4B (KDM4B) through its DUB activity, thereby promoting angiogenesis [38]. Similarly, UCHL1 has been shown to facilitate cell proliferation, migration, and invasion by activated protein kinase B (Akt) and extracellular signal-regulated kinase 1/2 (Erk1/2) pathways in transforming growth factor-β (TGFβ)-induced breast cancer metastasis [39]. Although the key transcription factor involved in EMT, Twist1, has been demonstrated to promote EMT and metastasis in NSCLC while conferring resistance to chemotherapy [9], the precise mechanism through which UCHL1 mediates NSCLC metastasis through its deubiquitinase activity, as well as the specific posttranslational modification processes involved, has remained elusive. In this study, we confirmed the interaction between UCHL1 and Twist1 and demonstrated that UCHL1 modulates the stability of Twist1 via its DUB activity. Protein ubiquitination typically involves the attachment of ubiquitin chains to the substrate protein, with K11- and K63-linked chains being implicated in the degradation of substrate proteins and the execution of their biological functions [40, 41]. Our data indicate that UCHL1 regulates the stability of Twist1 by cleaving its K11- and K63-linked ubiquitin chains. These results are in line with previous findings that the inhibition of UCHL1 results in an increase in the abundance of K63-linked ubiquitin chains in oocytes [42].

Nonetheless, our research has certain limitations. First, when the relationship between UCHL1 expression and metastasis in NSCLC was explored, the number of samples for immunohistochemistry was insufficient. The residues on Twist1 that are modified by K11- and K63-linked ubiquitin chains mediated by UCHL1 still need to be further identified. Second, the mechanism through which Twist1 deubiquitination affects EMT needs to be further studied in the future. It is unclear whether UCHL1 only regulates the stability of Twist1 or may also regulate its nuclear localization or transcriptional activity. Third, despite the advantages of the zebrafish model, such as its optical transparency and rapid experimental turnaround, several limitations must be acknowledged. The primary constraints include a relatively narrow observation window, which restricts long-term studies of tumor cell proliferation and colonization. Furthermore, the zebrafish tumor microenvironment cannot fully recapitulate the complexity of its mammalian counterpart, potentially limiting the translational relevance of certain findings. We will conduct further research on the regulatory effects of UCHL1 on Twist1 in EMT in the future.

In summary, our research revealed a novel mechanism through which UCHL1 exerts its tumor-promoting function in NSCLC metastasis. We demonstrated that UCHL1 facilitates NSCLC cell migration and EMT by directly interacting with Twist1 via its M1 region and cleaving the K11- and K63-linked ubiquitin chains of Twist1. The results of this study suggest that the UCHL1/Twist1 axis is a promising target for the development of new therapeutic interventions for NSCLC.

## Materials and methods

### Clinical samples

Tissue samples of primary and metastatic NSCLC were collected from patients at the Suzhou Municipal Hospital, Jiangsu, China, from January 2017 to December 2018, following the acquisition of informed consent. Prior to surgery, none of the patients had undergone chemotherapy or radiotherapy. The experimental protocols were reviewed and approved by the Ethics Committee of Suzhou Municipal Hospital, and all procedures were carried out in strict adherence to the principles outlined in the Helsinki Declaration.

### Immunohistochemistry

For immunostaining, an ABC (avidin-biotin complex) detection system (Benchmark, Angleton, Texas, USA) was utilized, adhering strictly to the manufacturer’s protocol [43]. The primary antibodies deployed in this study comprised anti-UCHL1 (13179, Cell Signaling Technology, MA, USA) and anti-Twist1 (131127, Absin, Shanghai, China). Negative control slides, which were not subjected to primary antibodies, exhibited no nonspecific staining, thereby validating the specificity of the immunostaining. Each slide was independently assessed by two pathologists in a blinded fashion to ensure an unbiased and objective evaluation.

### Western blotting (WB)

Western blotting analysis was performed following the available protocol [44]. The primary antibodies used in this study included anti-UCHL1 (13179, Cell Signaling Technology), anti-Twist1 (131127, Absin), anti-E-cadherin (14472, Cell Signaling Technology), anti-HA tag (3724, Cell Signaling Technology), anti-Ubiquitin (3933, Cell Signaling Technology), anti-Myc tag (2276, Cell Signaling Technology), anti-DDDDK tag (14793, Cell Signaling Technology), and anti-Vimentin (131996, Absin). Following incubation with primary antibodies, the membranes were then incubated with DyLight 800-conjugated rabbit or mouse secondary antibodies (KPL). The immunoreactive bands were visualized and scanned using an Odyssey Infrared Imaging System (LI-COR). Additionally, full and uncropped western blots have been uploaded as ‘Supplemental Material’.

### Quantitative real-time PCR (qRT-PCR)

Total RNA was extracted from cultured cells using the TRIzol reagent (Thermo Scientific, MA, USA). Subsequently, RNA was reverse transcribed into cDNA using the FastKing gDNA Dispelling RT SuperMix (Tiangen, Beijing, China). qRT**-**PCR was performed using the FastFire qPCR Pre Mix (SYBR Green) with species-specific primers. Each experiment was biologically replicated three times. GAPDH was used as the internal control, and mRNA levels of all samples were normalized to those of GAPDH. Primer sequences are listed in Table S1.

### Immunoprecipitation

Immunoprecipitation was performed according to a standard protocol [45]. Briefly, 4 × 10^6^ cells were lysed for 30 min at 4 °C with gentle rotation in 1 mL of lysis buffer containing 50 mM Tris-HCl (pH 8.0), 150 mM NaCl, 1% NP**-**40, and a protease inhibitor cocktail (Roche, Basel, Switzerland). The clarified cell lysate was then mixed with 1 μg of the specific antibody and incubated overnight at 4 °C with slow rotation. Following this, the samples were added to 20 μL of Protein A/G Plus Agarose Beads (Sigma-Aldrich, St. Louis, MO) and rotated for an additional 3 h at 4 °C. The beads were washed three times with phosphate-buffered saline (PBS) supplemented with 0.5% Tween 20. Proteins bound to the beads were subsequently heat-eluted in 80 μL of SDS**-**PAGE reducing sample buffer and analyzed by western blotting using the appropriate antibodies.

### Cell lines and culture conditions

NSCLC cell lines (H358, PC-9, A549, H1299, and 95-D) were obtained from the Type Culture Collection of the Chinese Academy of Sciences (Shanghai, China), and cultured in a humidified atmosphere containing 5% CO_2_ at a controlled temperature of 37 °C.

### Lentivirus transfection and isolation of stable cell clones

HEK293T cells were employed to generate recombinant lentiviral particles through the cotransfection of the transfer Vector with packaging plasmids pMD2.G (Addgene, Cambridge, UK) and pSPAX2 (Addgene) using Lipofectamine 2000 (Invitrogen, Carlsbad, CA, USA), adhering strictly to the manufacturer’s guidelines. Lentiviruses encoding short hairpin RNA (shRNA) were subsequently produced and utilized to infect NSCLC cells in the presence of polybrene (Life Technologies, Darmstadt, Germany), following the manufacturer’s recommended protocol. The culture medium was refreshed 24 h postinfection. Subsequently, the medium was replaced with RPMI-1640, supplemented with 10% fetal bovine serum (FBS) and 2 μg/mL puromycin (MedChemExpress, Monmouth Junction, NJ, USA), to facilitate the selection of transduced cells. Once puromycin resistance was established, individual clones from each transfection group were isolated and evaluated for UCHL1 protein knockdown via western blotting analysis.

### Cell migration assay

The cell migration assay was performed according to an established protocol [46]. For the wound-healing assay, NSCLC cells were seeded into 6-well plates and cultured until a confluent monolayer was achieved. A sterile 200-μL pipette tip was then employed to create a linear scratch across the cell layer, thereby inducing a wound. After wounding, the culture was replenished with fresh medium containing 1% FBS and further incubated. The progression of wound closure was monitored by calculating the ratio of the wound width at 24 h to that at the initial time point (0 h). The migratory capacity of the NSCLC cells was evaluated by observing cellular movement into the denuded area under an inverted phase contrast microscope.

In the transwell assay, NSCLC cells subjected to various treatments were seeded into the upper chamber of a transwell insert. The lower chamber was filled with complete growth medium. Following a 37 °C incubation period of 3 h, the non-migrated cells on the inner surface of the inserts were meticulously removed, and the membranes were washed three times with PBS. The membranes were then fixed with 4% paraformaldehyde (PFA) and stained with crystal violet. The number of migrated cells was quantified by capturing images of the stained membranes under a microscope and counting the cells accordingly.

### Immunofluorescence staining

Cells were cultured on glass coverslips and subsequently fixed with 4% PFA for 20 min. Following fixation, the cells were rinsed with PBS and permeabilized using 0.1% Triton X-100. To mitigate nonspecific binding, the cells were blocked with 2% bovine serum albumin (BSA) prior to an overnight incubation at 4 °C with primary antibodies targeting UCHL1 (13179, Cell Signaling Technology), Twist1 (131127, Absin), and Ki67 (ab15580, Abcam, MA, USA). On the subsequent day, the cells were incubated for 1 h at 37 °C in the dark with Alexa Fluor 488-conjugated secondary antibody (A-11008, Invitrogen) to visualize primary antibody binding. Additionally, 4′,6-diamidino-2-phenylindole (DAPI, D9542, Sigma-Aldrich, St. Louis, MO, USA) was employed to stain cell nuclei. Upon completion of immunofluorescence labeling, the coverslips were mounted onto microscope slides using a fluorescent mounting medium. The prepared slides were then immediately visualized under a fluorescence microscope (Carl Zeiss, Jena, Germany).

### Tumor growth in nude mice

To investigate the role of UCHL1 in metastasis in vivo, a lung cancer metastasis model was established through tail-vein injection of lung cancer cells. For assessing the influence of UCHL1 on lung metastasis, mice were divided into two experimental cohorts: one injected with luciferase/Vector cells and the other with luciferase/*UCHL1* cells, each at a density of 5 × 10^6^ cells/mL. Each cohort comprised four animals. The cells were administered via tail-vein injection. The progression of cancer in the lungs was monitored over a 42-day period using bioluminescent imaging facilitated by the IVIS100 Imaging System (Kodak, Rochester, NY, USA).

### Zebrafish xenograft tumor model

Zebrafish were reared, staged, and maintained in accordance with established standard procedures. All zebrafish experiments were carried out with the approval of the Nanjing Medical University’s Laboratory Animal Management and Use Committee. At 48 h post-fertilization, embryos were immobilized on a low-melting-point agarose plate for experimental manipulation. A suspension of H1299 cells, pre-stained with CellTracker™ CM-DiI dye (C7000, Invitrogen), was injected into the perivitelline space of the zebrafish embryos, with each injection containing 400 cells. Six days post-injection, the zebrafish were fixed using 4% PFA. Imaging and subsequent analysis of the results were conducted using an inverted confocal microscope (Leica). For each experimental group, a minimum of 10 zebrafish were examined, and three representative images were captured. The experiments were replicated at least three times, and the results presented are indicative of the consistent findings observed.

### Statistical analysis

Data are presented as the mean ± standard error of the mean (SEM), derived from three independent experiments. Statistical analyses were performed using GraphPad Prism 6.0 software. Statistical comparisons between two groups were conducted using Student’s *t*-test, whereas for comparisons involving three or more groups, ordinary one-way analysis of variance (ANOVA) was utilized. To assess the correlation between the expression of two proteins, Spearman’s rank correlation analysis was conducted. The statistical significance of Kaplan–Meier survival curves was evaluated using the log-rank test. A *p*-value of less than 0.05 was considered indicative of statistical significance.

## Supplementary information

Supplementary Figure

Supplementary Table

Full and uncropped western blots

## Data Availability

Additional data generated in this study are available upon request from the corresponding author.
