# Supplementary material for: UCHL1 stabilizes Twist1 via K11/K63-linked deubiquitination to drive tumor metastasis in non-small cell lung cancer
Source: Cell Death Discov. 2025 Dec 30;12:60. doi: 10.1038/s41420-025-02925-8 (PMC12847959; doi:10.1038/s41420-025-02925-8)
Supplement: Supplementary file 1 — Supplementary Figure [file 41420_2025_2925_MOESM1_ESM.docx]

**SUPPLEMENTARY FIGURES**


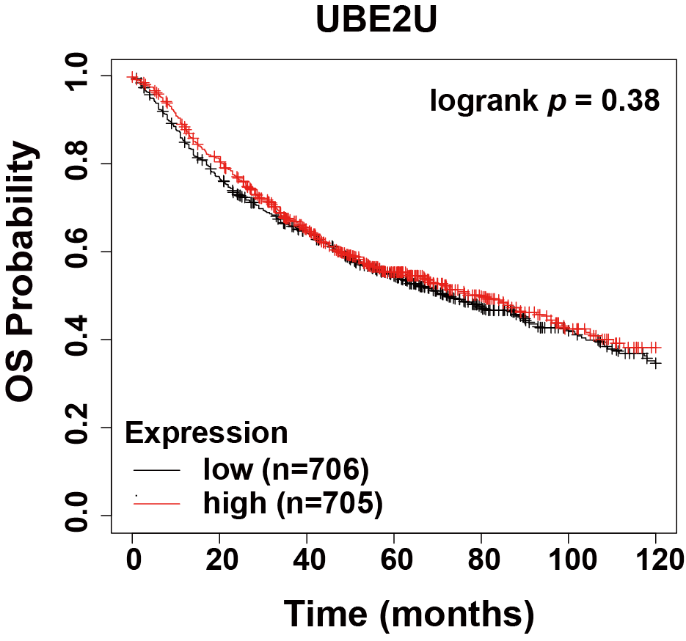


**Fig. S1** Overall survival (OS) was analyzed based on the expression of *UBE2U* in NSCLC samples using Kaplan–Meier plotter.


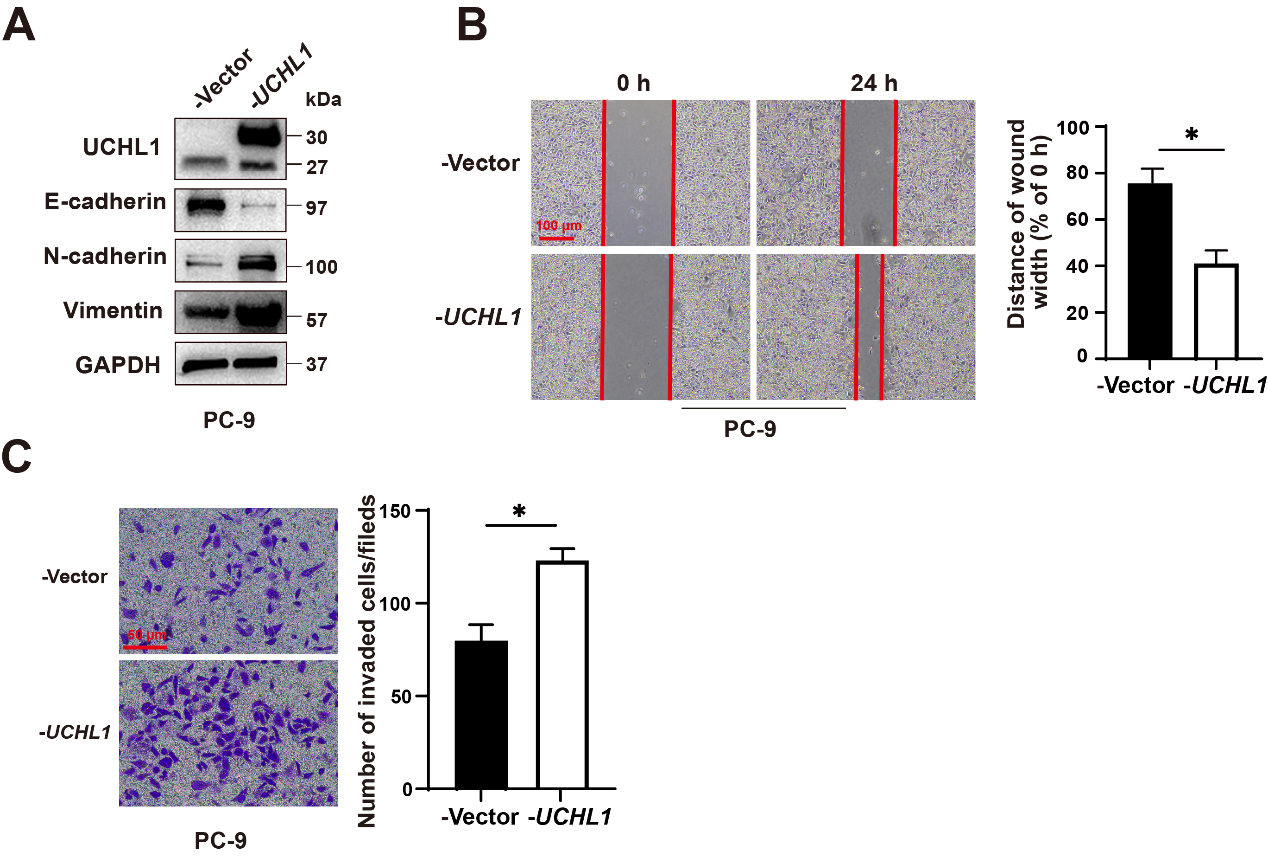


**Fig. S2 A** EMT marker protein levels were assessed in PC-9 cells transfected with vector or Flag-*UCHL1* plasmids by western blotting. **B** Cell migration was determined using wound-healing assays. Red bar, 100 μm. **C** Cell invasion was evaluated in a Transwell invasion assay. Red bar, 50 μm. **p* < 0.05.


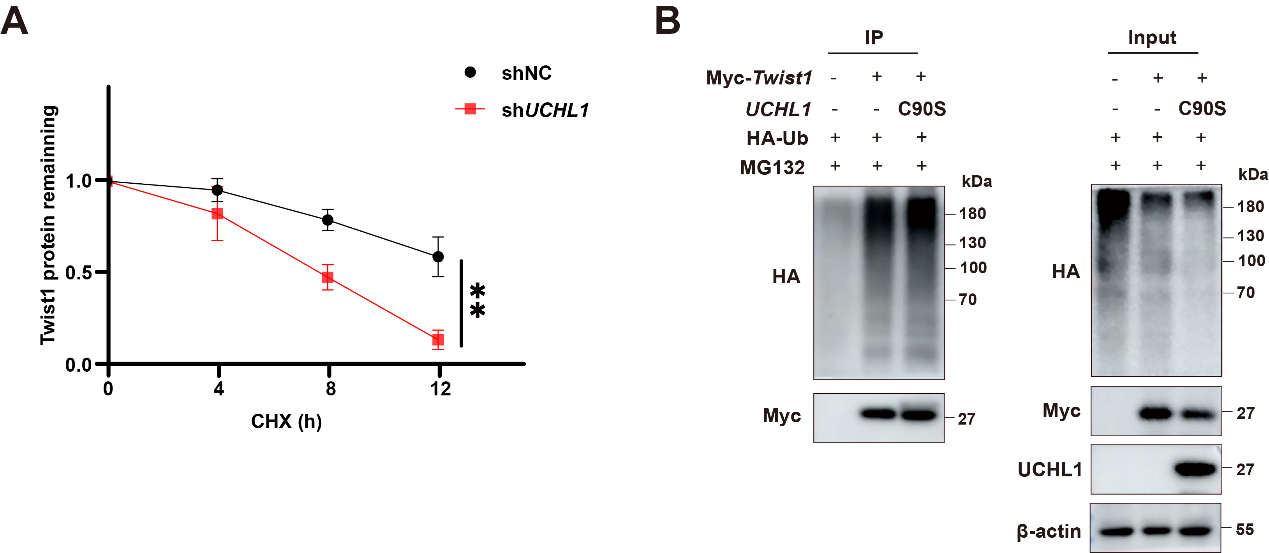


**Fig. S3** **A** Half-life analysis of Twist1 abundance in shNC or sh*UCHL1* cells for the indicated time periods in the presence of CHX. Data are shown as mean ± SEM of 3 independent experiments. two-way ANOVA test, ***p* < 0.01. **B** HEK293T cells that were cotransfected with Myc-*Twist1*, HA-Ub and UCHL1-C90S constructs were treated with 20 μM MG132 for 8 hours, and the ubiquitination level of Myc-Twist1 was assessed using ubiquitination assays.


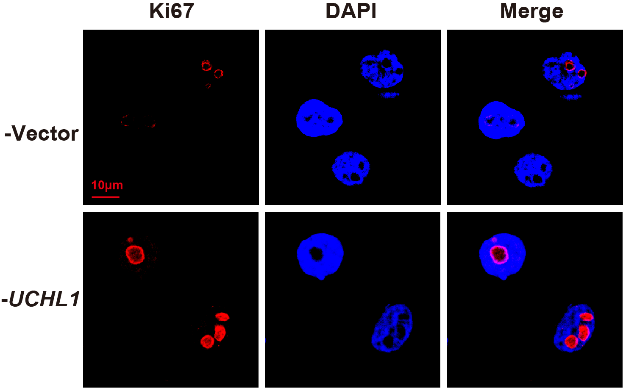


**Fig. S4** Ki67 staining in PC-9 cells was visualized by confocal microscopy, with nuclei counterstained with DAPI. Red bar, 10 μm.
