# Supplementary material for: UCHL1 stabilizes Twist1 via K11/K63-linked deubiquitination to drive tumor metastasis in non-small cell lung cancer
Source: Cell Death Discov. 2025 Dec 30;12:60. doi: 10.1038/s41420-025-02925-8 (PMC12847959; doi:10.1038/s41420-025-02925-8)
Supplement: Supplementary file 2 — Supplementary Table [file 41420_2025_2925_MOESM2_ESM.docx]

**Supplementary Table**

**Table S1. RT-qPCR primers**

| **Gene symbol** | **Sequences (5’ - 3’)** |
| --- | --- |
| Human *UCHL1* | CCTGTGGCACAATCGGACTTA CATCTACCCGACATTGGCCTT |
| Human *TWIST1* | GTCCGCAGTCTTACGAGGAG GCTTGAGGGTCTGAATCTTGCT |
| Human *GAPDH* | GGAAGATGGTGATGGGATT GGATTTGGTCGTATTGGG |
