# Supplementary material for: UCHL1 stabilizes Twist1 via K11/K63-linked deubiquitination to drive tumor metastasis in non-small cell lung cancer
Source: Cell Death Discov. 2025 Dec 30;12:60. doi: 10.1038/s41420-025-02925-8 (PMC12847959; doi:10.1038/s41420-025-02925-8)
Supplement: Supplementary file 3 — Full and uncropped western blots [file 41420_2025_2925_MOESM3_ESM.pdf]

Figure 2A

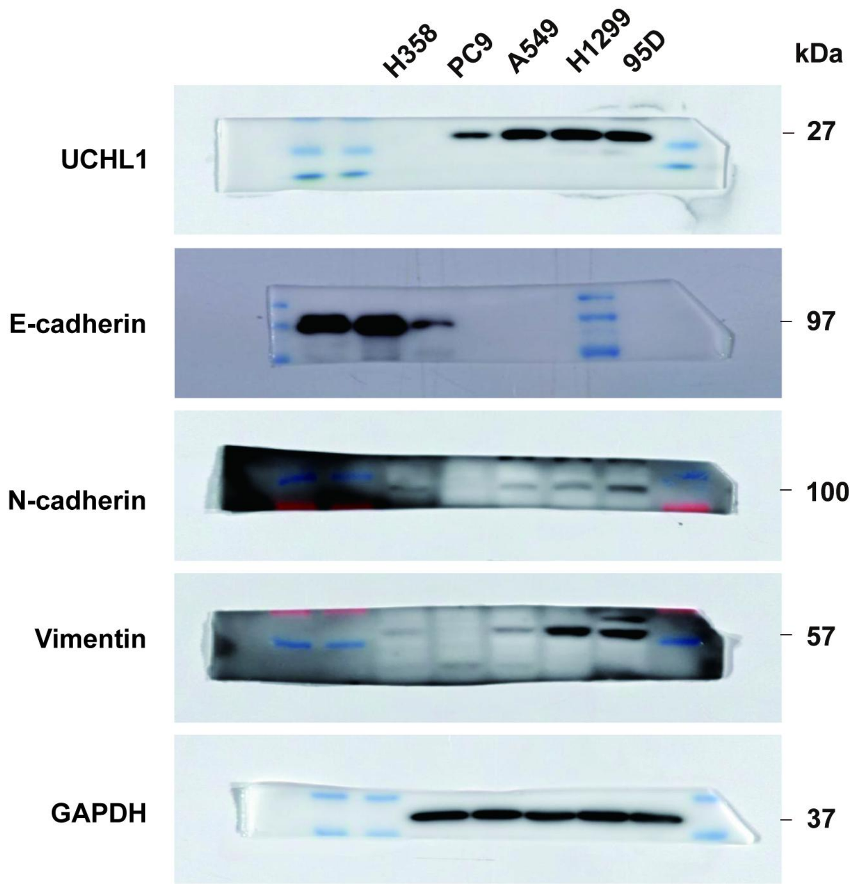
H358
PC9
A549
H1299
95D
kDa
UCHL1
27
E-cadherin
97
N-cadherin
100
Vimentin
57
GAPDH
37

Figure 2D

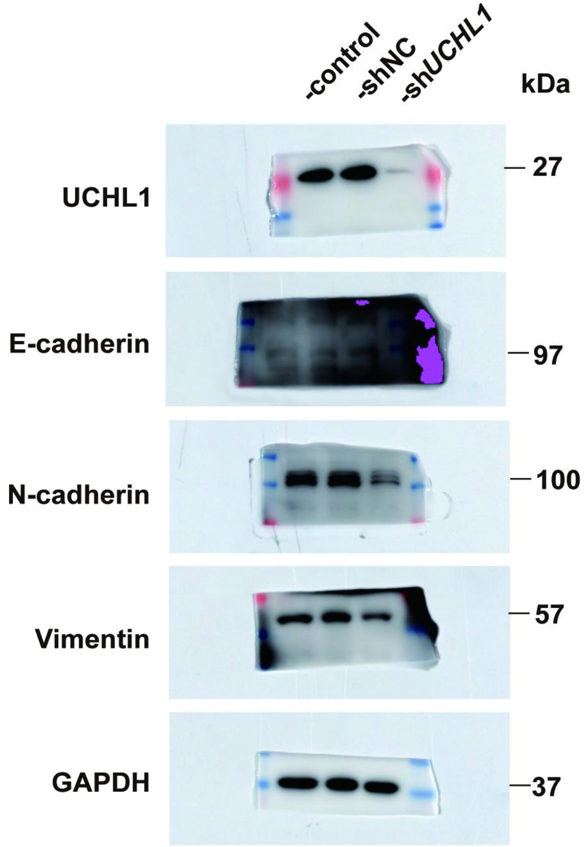

-control
-shNC
-shUCHL1
kDa
UCHL1
27
E-cadherin
97
N-cadherin
100
Vimentin
57
GAPDH
37

Figure 2E

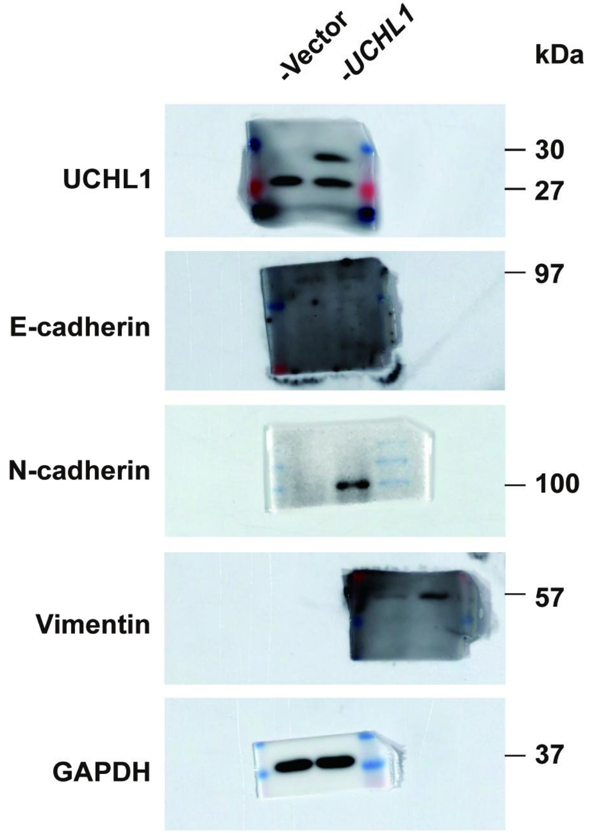

-Vector
-UCHL1
kDa
UCHL1
30
27
E-cadherin
97
N-cadherin
100
Vimentin
57
GAPDH
37

Figure 3A

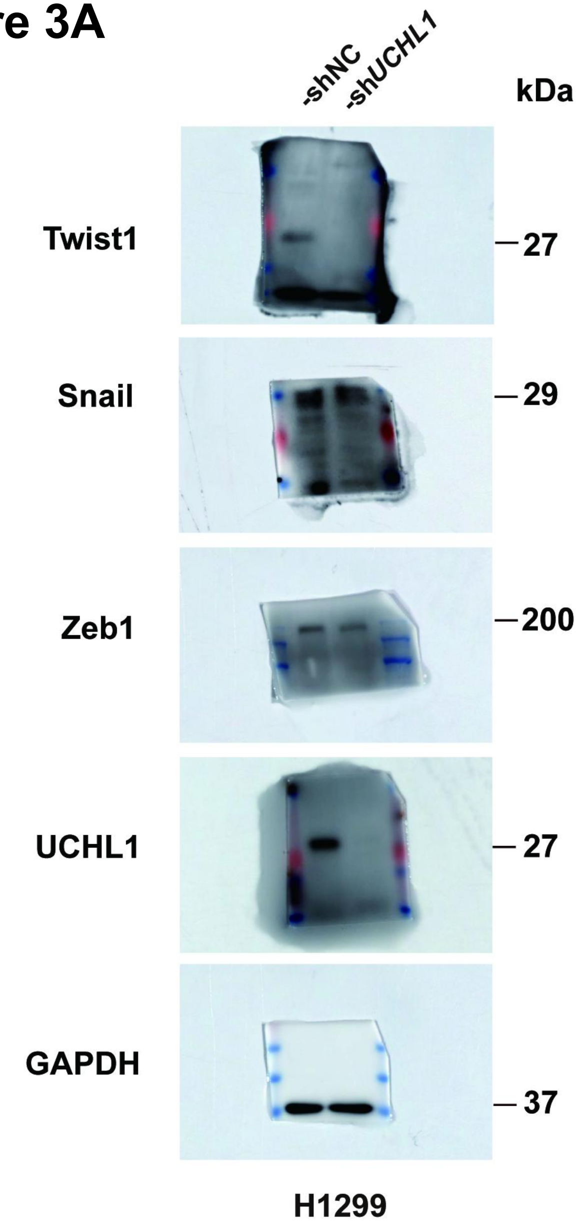
-shNC
-shUCHL1
kDa
Twist1
27
Snail
29
Zeb1
200
UCHL1
27
GAPDH
37
H1299

Figure 3B

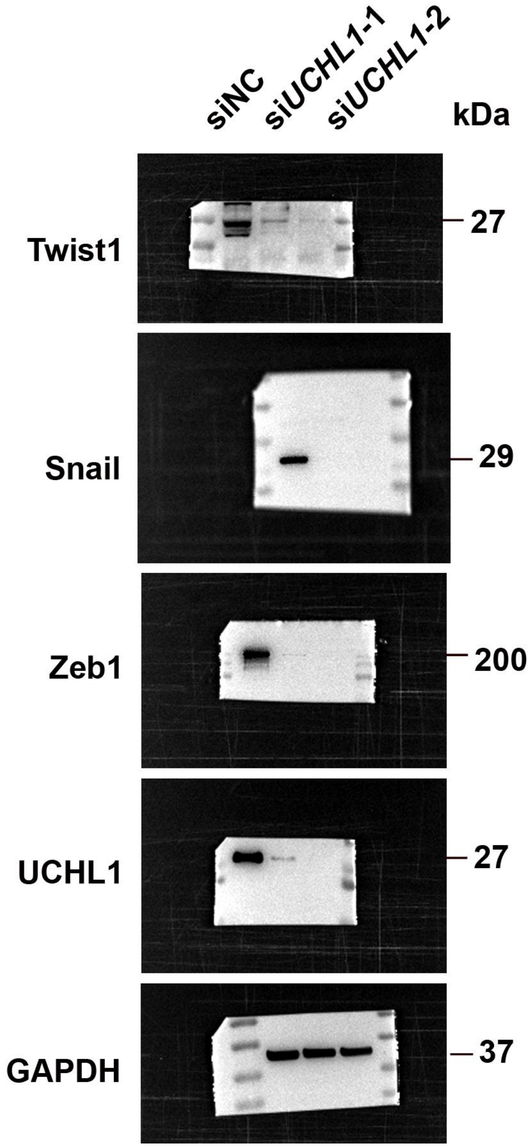
siNC
siUCHL1-1
siUCHL1-2
kDa
Twist1
27
Snail
29
Zeb1
200
UCHL1
27
GAPDH
37

Figure 3C

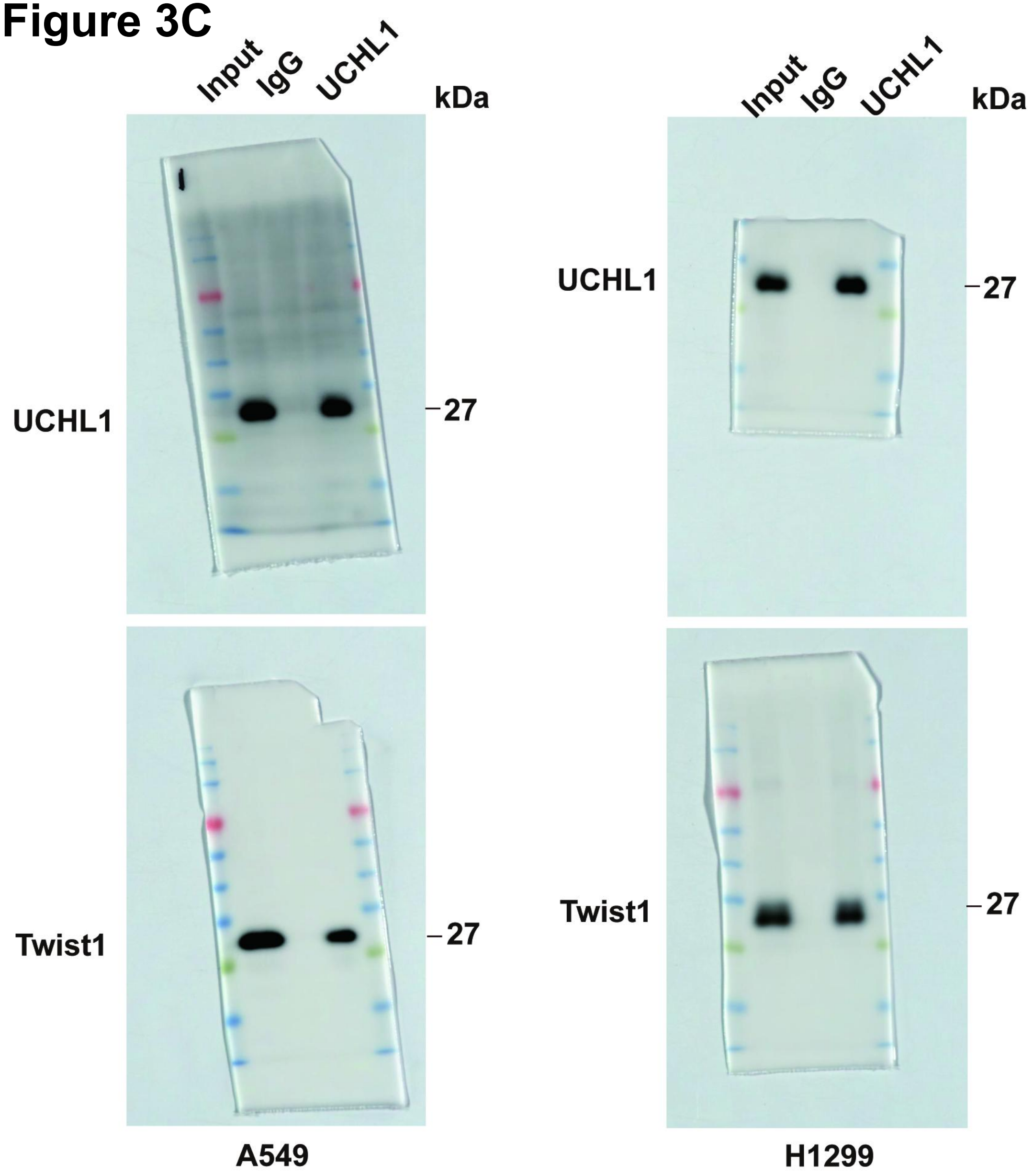

Input
IgG
UCHL1
kDa
UCHL1
27
Twist1
27
A549
Input
IgG
UCHL1
kDa
UCHL1
27
Twist1
27
H1299

Figure 3F

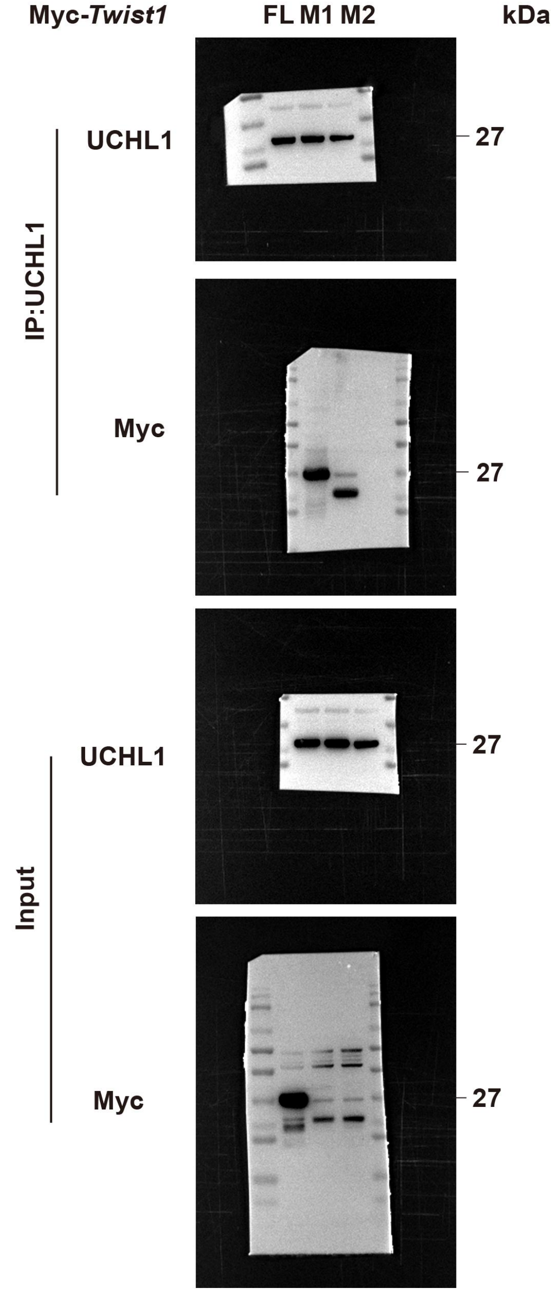

Myc-Twist1
FL M1 M2
kDa
IP:UCHL1
UCHL1
27
Myc
27
Input
UCHL1
27
Myc
27

Figure 3H

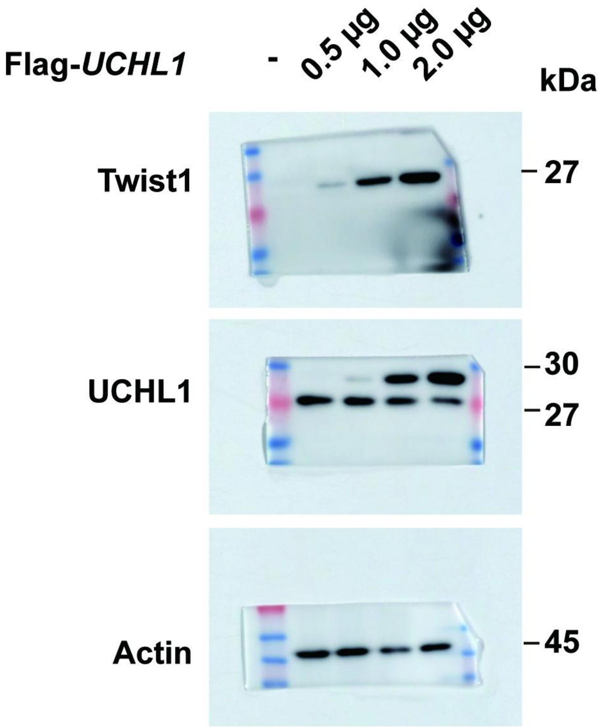

Figure 4A

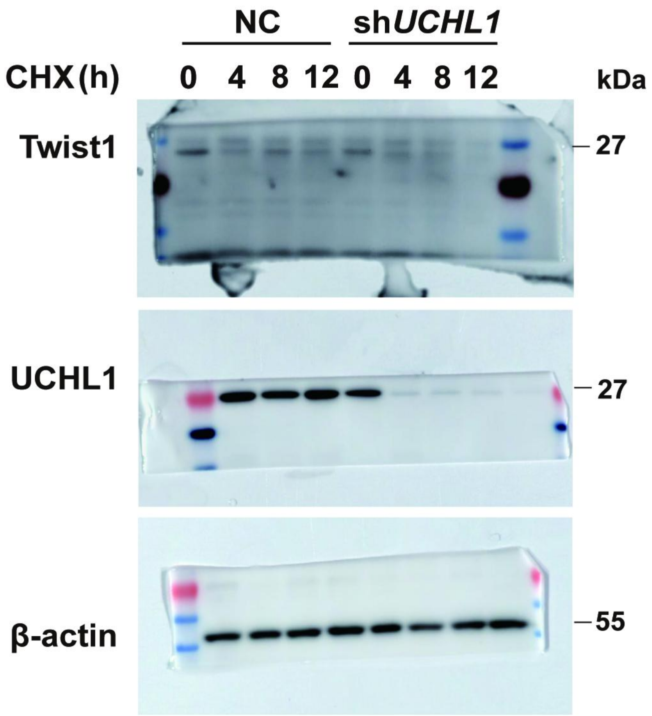

Figure 4B

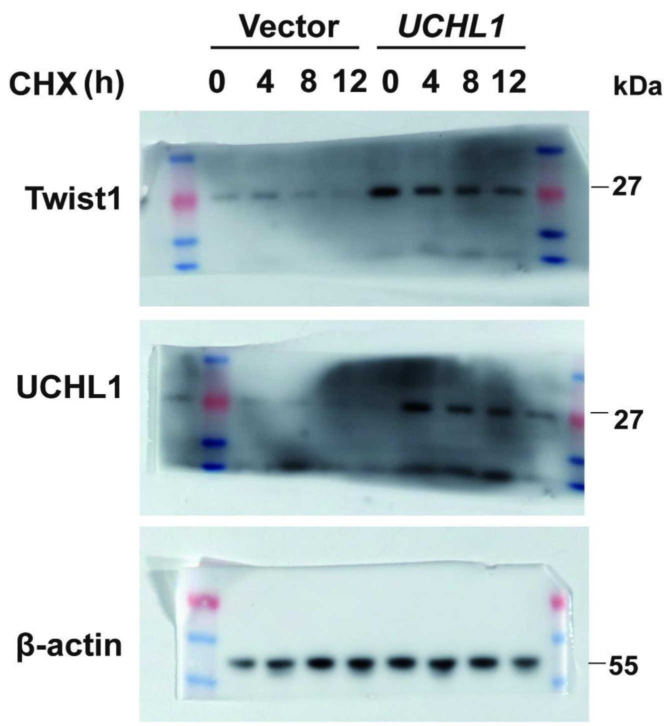

Vector
UCHL1
CHX (h)
0 4 8 12 0 4 8 12
kDa
Twist1
27
UCHL1
27
β-actin
55

Figure 4C

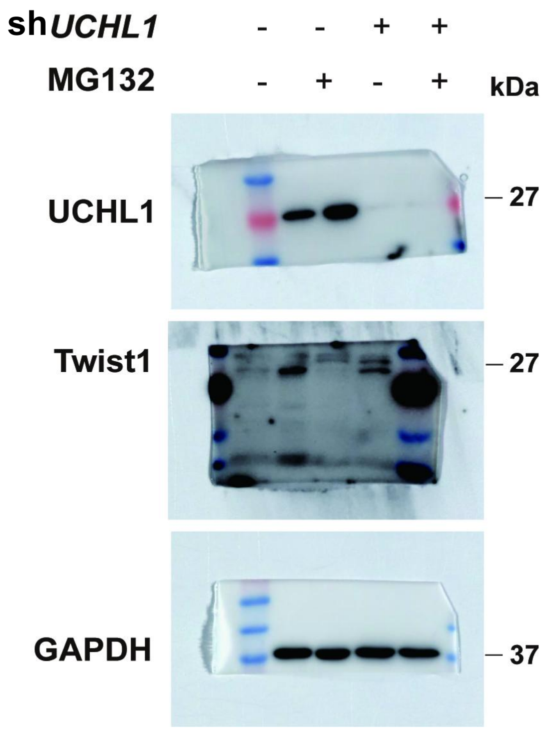

Figure 4D

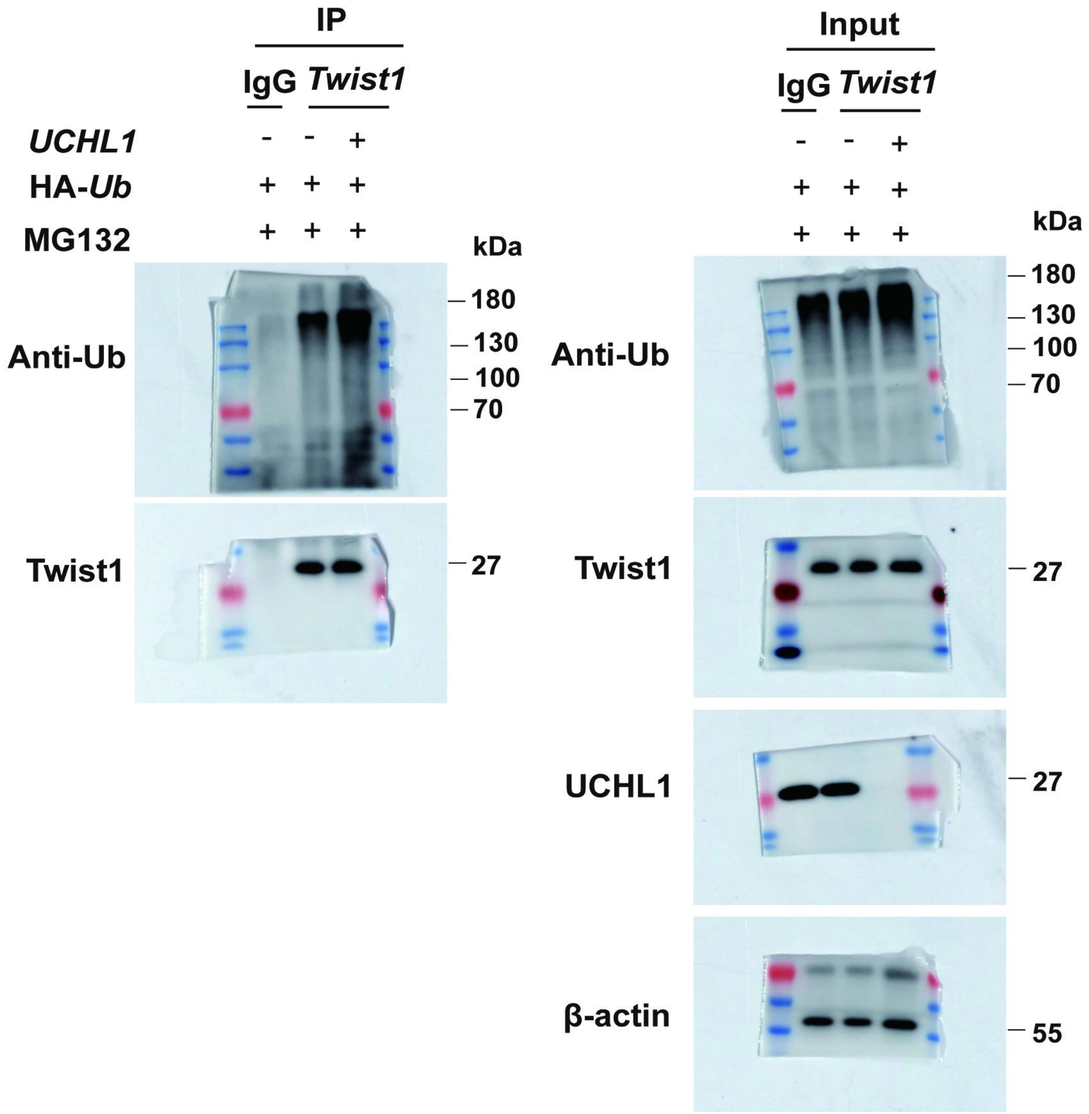
IP
IgG
Twist1
UCHL1
HA-Ub
MG132
- - +
+ + +
+ + +
kDa
Anti-Ub
180
130
100
70
Twist1
27
Input
IgG
Twist1
- - +
+ + +
+ + +
kDa
Anti-Ub
180
130
100
70
Twist1
27
UCHL1
27
β-actin
55

Figure 4E

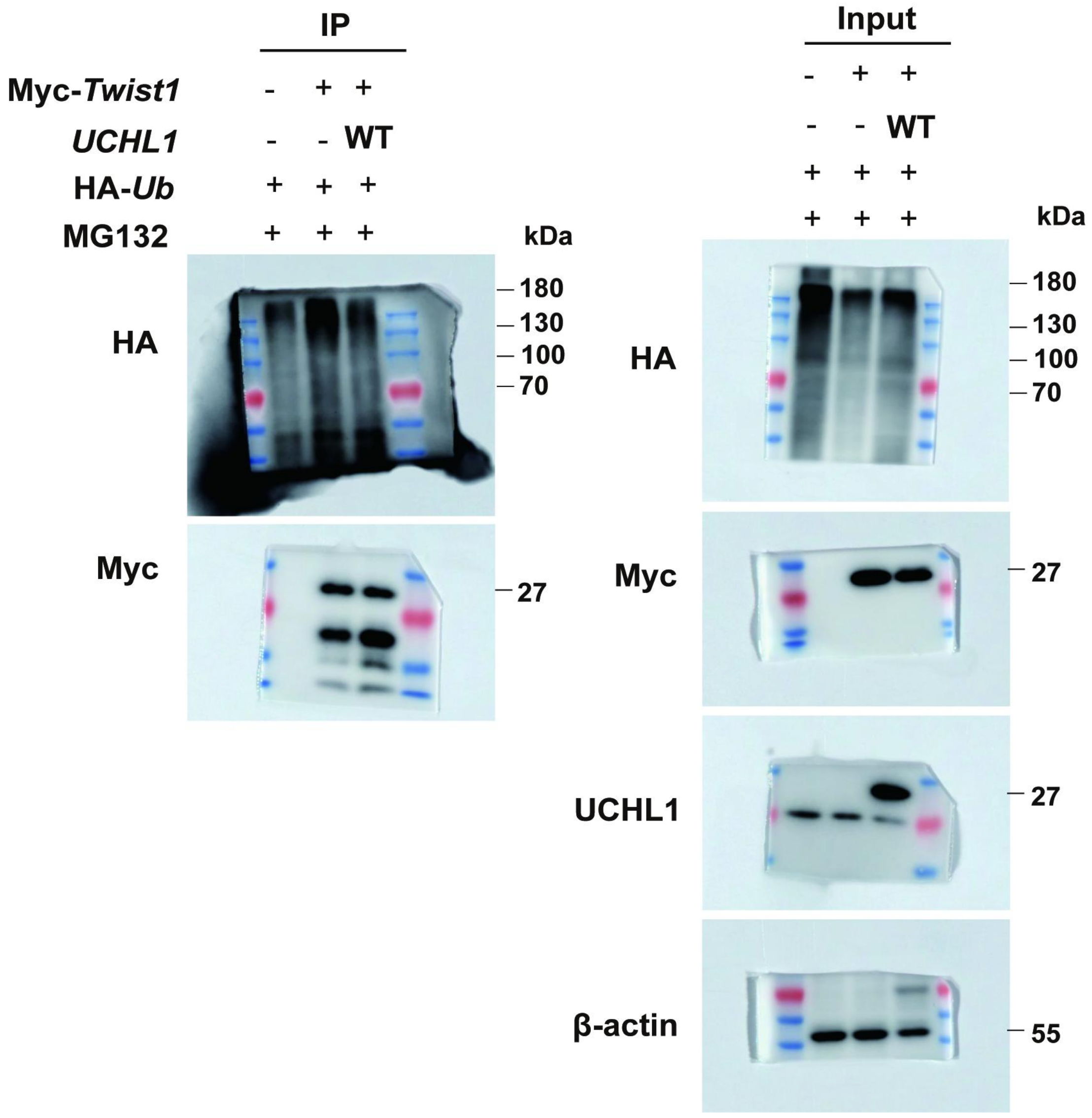
IP
Input
Myc-Twist1
UCHL1
HA-Ub
MG132
- + +
- - WT
+ + +
+ + +
kDa
HA
180
130
100
70
Myc
27
UCHL1
27
β-actin
55

Figure 4F

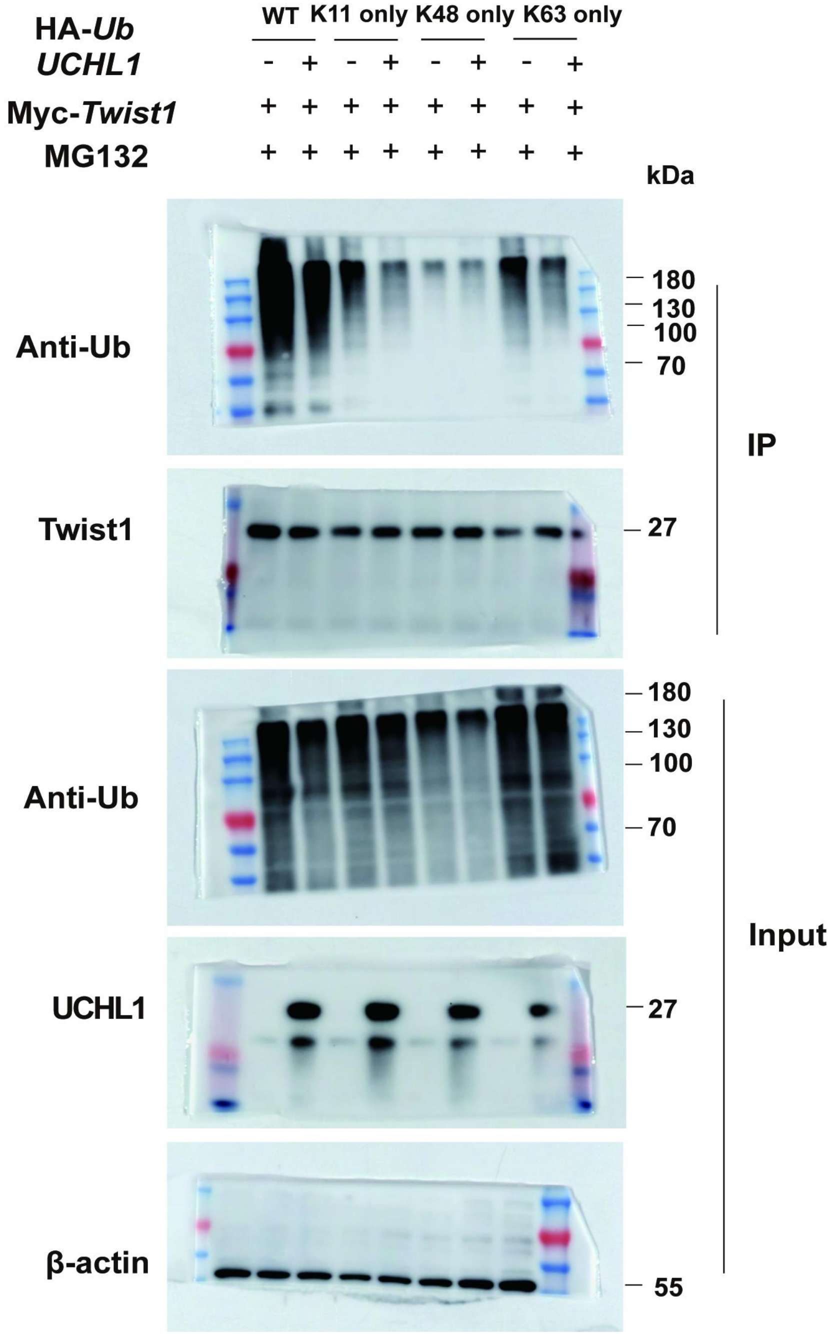
HA-*Ub*
WT
K11 only
K48 only
K63 only
UCHL1
- + - + - + - +
Myc-*Twist1*
+ + + + + + + +
MG132
+ + + + + + + +
kDa
Anti-Ub
180
130
100
70
Twist1
27
IP
Anti-Ub
180
130
100
70
UCHL1
27
Input
β-actin
55

Figure 4G

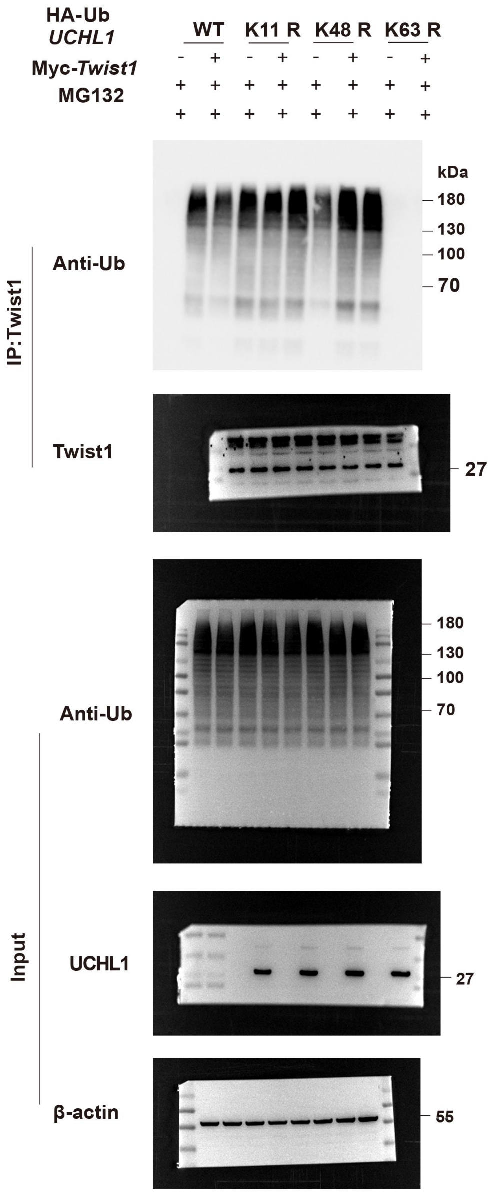

HA-Ub
UCHL1
Myc-Twist1
MG132
WT
K11 R
K48 R
K63 R
kDa
180
130
100
70
IP:Twist1
Anti-Ub
Twist1
27
Input
Anti-Ub
180
130
100
70
UCHL1
27
β-actin
55

Figure 5A

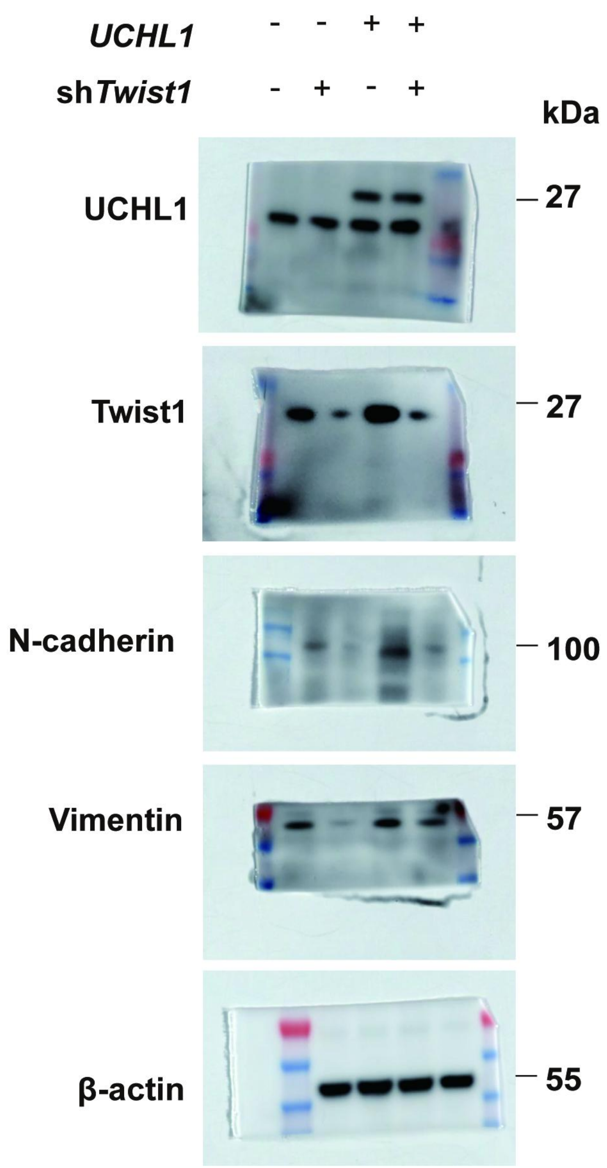
UCHL1 - - + +
shTwist1 - + - +
kDa
UCHL1
27
Twist1
27
N-cadherin
100
Vimentin
57
β-actin
55

Figure S2A

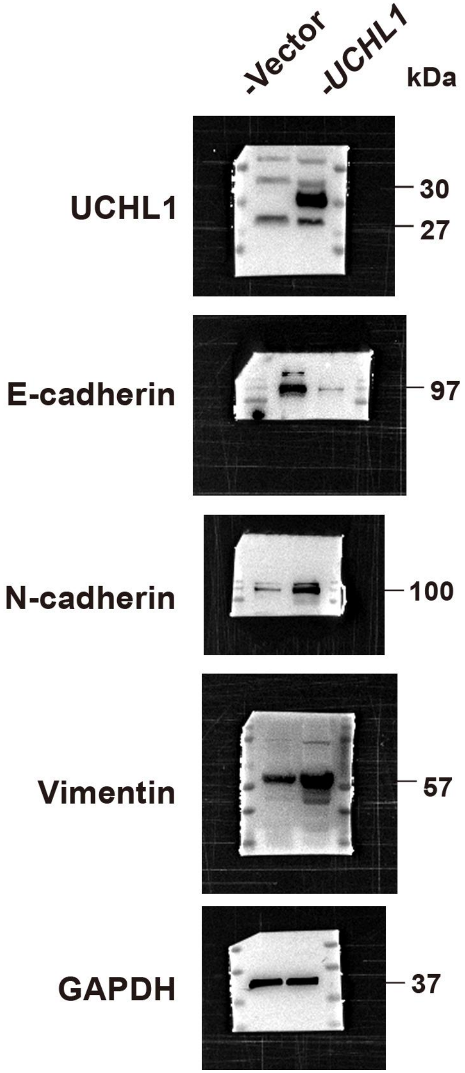
-Vector
-UCHL1
kDa
UCHL1
30
27
E-cadherin
97
N-cadherin
100
Vimentin
57
GAPDH
37

Figure S3B

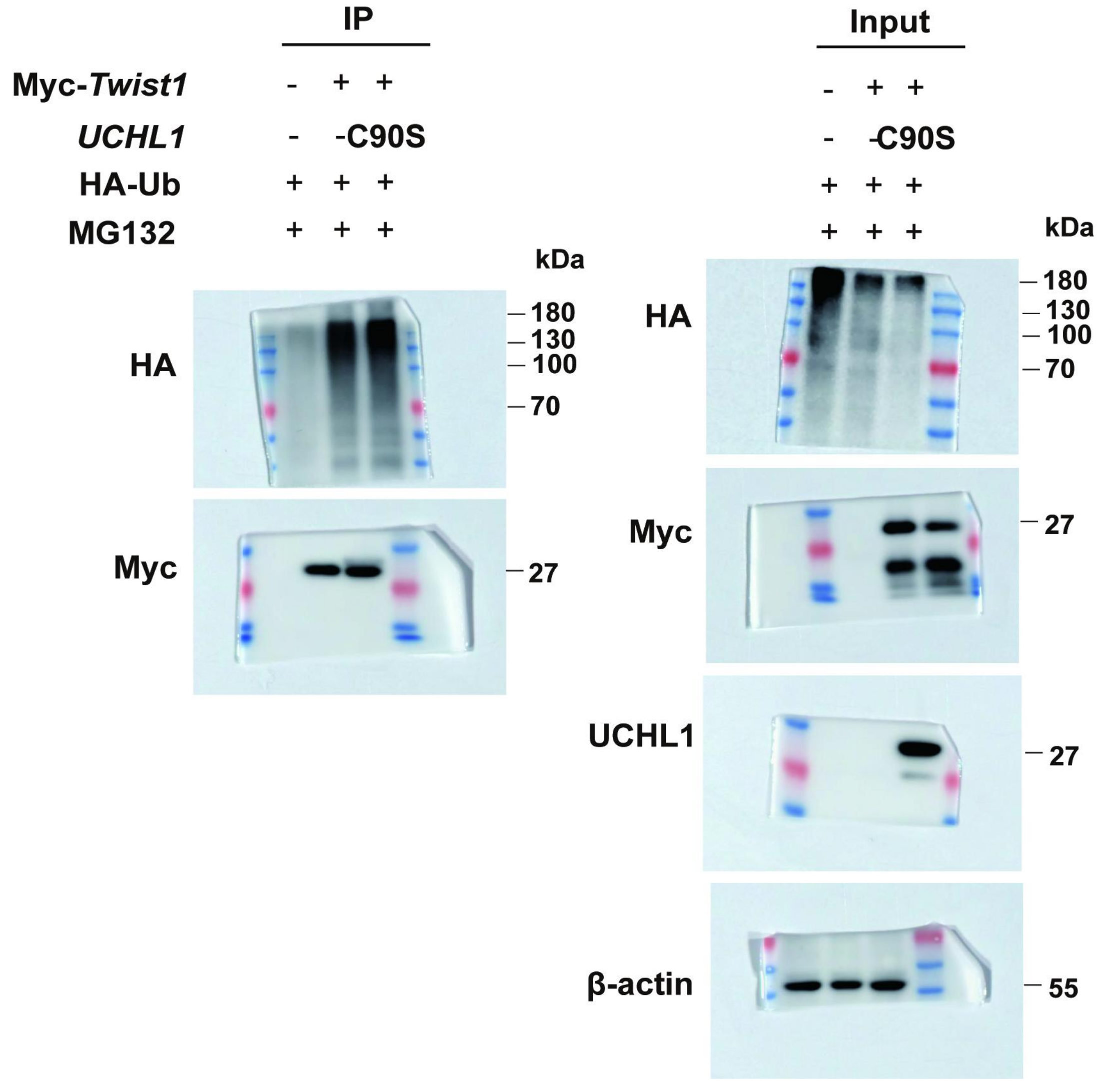
IP
Input
Myc-*Twist1*
- + +
UCHL1
- - C90S
HA-Ub
+ + +
MG132
+ + +
kDa
HA
180
130
100
70
Myc
27
UCHL1
β-actin
55
